# epiG: statistical inference and profiling of DNA methylation from whole-genome bisulfite sequencing data

**DOI:** 10.1186/s13059-017-1168-4

**Published:** 2017-02-21

**Authors:** Martin Vincent, Kamilla Mundbjerg, Jakob Skou Pedersen, Gangning Liang, Peter A. Jones, Torben Falck Ørntoft, Karina Dalsgaard Sørensen, Carsten Wiuf

**Affiliations:** 1 Department of Mathematical Sciences, University of Copenhagen, Copenhagen, 2100 Denmark; 2 USC Norris Comprehensive Cancer Center, Keck School of Medicine, Los Angeles, 90089-9176 CA USA; 3 Department of Molecular Medicine, Aarhus University Hospital, Aarhus, 8200 Denmark; 4 Department of Urology, Keck School of Medicine, University of Southern California, Los Angeles, 90089 CA USA; 5 Van Andel Research Institute, Grand Rapids, 49503 MI USA

**Keywords:** CpG methylation, GpC methylation, NOMe-seq, Epi-allelic haplotype, Epi-allele, Epigenetic state

## Abstract

The study of epigenetic heterogeneity at the level of individual cells and in whole populations is the key to understanding cellular differentiation, organismal development, and the evolution of cancer. We develop a statistical method, epiG, to infer and differentiate between different epi-allelic haplotypes, annotated with CpG methylation status and DNA polymorphisms, from whole-genome bisulfite sequencing data, and nucleosome occupancy from NOMe-seq data. We demonstrate the capabilities of the method by inferring allele-specific methylation and nucleosome occupancy in cell lines, and colon and tumor samples, and by benchmarking the method against independent experimental data.

**Electronic supplementary material:**

The online version of this article (doi:10.1186/s13059-017-1168-4) contains supplementary material, which is available to authorized users.

## Background

The epigenetic state of a particular genomic region is the combined configuration of the epigenetic modifications in the region, including DNA (CpG) methylation, nucleosome positioning, and histone modifications. Such states are important regulators of many biological processes in health and disease, and at many different biological levels, for example gene regulation, cellular differentiation, and organismal development [1–3]. To understand the biological consequences of CpG methylation and nucleosome positioning, it is, therefore, important to be able to extract reliable epigenetic information from genome-wide sequencing data using statistical methods.

A particularly challenging problem for statistical epigenetic inference is the possible heterogeneous nature of the epigenetic states [4, 5]. Heterogeneity occurs at the population level, for example, in relation to cellular development and differentiation [6], and in the evolution of cancer [7, 8]. It also occurs at the level of individual cells in the form of allele-specific methylation (ASM) [9–13]. ASM might stretch over many kilobases, and is associated with genomic imprinting [9, 14] and generally with genetic variants underlying phenotypic differences and complex diseases [13, 15]. Statistical methods that are able to call regions with ASM, and to infer and differentiate between different stable common epi-alleles in a biological sample, are therefore desirable. We have developed a stand-alone package, epiG, for this purpose.

The method epiG takes as input whole-genome bisulfite sequencing (WGBS) data [4, 16, 17]. It is a likelihood-based method that clusters reads into epi-allelic haplotypes based on sequence similarity, while taking into account experimental errors and biological noise. It outputs the dominating epi-allelic haplotypes of a genomic region of interest, annotated with an inferred CpG methylation and single nucleotide polymorphism (SNP)/somatic nucleotide variant (SNV) profile for each epi-allelic haplotype. The method makes use of prior information on base-calling quality, bisulfite conversion efficiency, and a reference SNP database. Recently, a protocol (NOMe-seq) for simultaneously assessing nucleosome positioning and CpG methylation genome-wide has been developed [18]. epiG has a NOMe-seq track that additionally performs inference on nucleosome occupancy.

State-of-the-art methods for WGBS methylation analysis infer CpG methylation position-wise, reporting a degree of methylation for each CpG site, e.g. [19–21]. Consequently, these methods do not allow for profiling of epigenetic haplotypes. Several methods exist for detecting differentially methylated regions, in particular with the aim of identifying ASM genes [9, 22, 23]. These methods, too, do not profile epigenetic haplotypes directly.

We demonstrate the applicability of epiG by inferring ASM and dominating epi-alleles in tumor and non-tumor WGBS samples. Also, we infer nucleosome positioning in two NOMe-seq samples from cell lines. By benchmarking with independent experimental data, we demonstrate that epiG calls CpG methylation states and genotypes correctly.

## Results and discussion

It is not straightforward to extract precise DNA methylation profiles from raw WGBS data due to the complexity of the bisulfite conversion process [17].

**Fig. 1:**
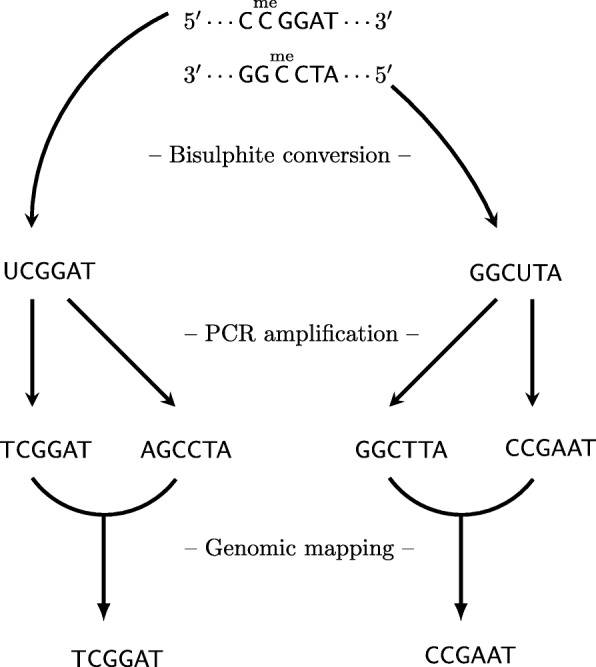
Bisulfite conversion. The DNA fragment is shown at the top as two complementary sequences. There are four possible base pairings of nucleotides and with C possibly methylated, this yields six different pairs in total, all of which are shown at the top of the figure. Bisulfite conversion converts the forward strand into the left sequence and the reverse strand into the right sequence, by changing unmethylated C into U and leaving methylated C as C. Subsequent PCR amplification results in four pairwise complementary types, where U is changed to T, which causes a change to A on the complementary strand. Alignment of the reads to a reference genome results in two types, which are both read in the forward direction. Comparing the two reads yields six possible pairs of nucleotides. If there are no sequencing and PCR errors, then these six (out of 16) are the only possible pairs of nucleotides that might be observed in reads from a single DNA fragment. Each pair in the observed reads, for example A- A, corresponds to a unique pair in the DNA fragment, here A- T

Figure 1 illustrates the conversion of genomic DNA into sequenced reads via bisulfite conversion, PCR amplification, and genomic mapping. For a single DNA fragment, the strand and the methylation status (top of Fig. 1) can be recovered from observed error-free reads (bottom of Fig. 1), as shown in Table 1. This is possible because an unmethylated C in a C- G site, with C on the forward strand, is converted into U by bisulfite conversion. After PCR amplification and genomic mapping, this results in an observed T- C site, that is, some reads with T and some reads with C in the matching position. Similarly, a G- C site with an unmethylated C becomes a G- A site after conversion, amplification, and mapping. Thus, the reads with T (G) unambiguously come from the forward strand, whereas the reads with C (A) unambiguously come from the reverse strand (see Table 1).

**Table 1:** Observed reads and the true epigenetic states

A	Read 1	T	C	G	G	A	T
	Read 2	C	C	G	A	A	T
	5^′^→3^′^	C	$\overset {\text {me}}{\mathsf {C}}$	G	G	A	T
	3^′^→5^′^	G	G	$\overset {\text {me}}{\mathsf {C}}$	C	T	A
B	Read		T	C	G	A	
	5^′^→3^′^		C/T	$\overset {\text {me}}{\mathsf {C}}/\mathsf {C}$	G	G/A	
	3^′^→5^′^		G/A	G	$\overset {\text {me}}{\mathsf {C}}/\mathsf {C}$	C/T	

The conditions for epigenetic inference are optimal if there are no sequencing and PCR errors. The table shows inference on the true epigenetic states from reads from a single DNA fragment

A. If error-free reads are available from both strands, one of six possible pairs of nucleotides might be observed at each site by comparing reads, as shown in the two top rows of the table (see also Fig. 1). In each case, the epigenetic state and the strand direction can be inferred unambiguously. For example, if G and A are observed, then the 5^′^→3^′^ strand has G, while the 3^′^→5^′^ strand has C unmethylated

B. If reads from only one strand are observed, then inference is in general inconclusive. For each of the four possible nucleotides that might be observed, the true epigenetic state and the strand direction cannot be inferred unambiguously. For example, if T is observed in a read, then the true epigenetic state of the fragment might be C- G or T- A, depending on whether the observed T comes from the 5^′^→3^′^ strand or the 3^′^→5^′^ strand. For correct epigenetic inference, it is, therefore, important that reads originating from both strands are observed

Our method is based on the above insight. The primary goal of epiG is to infer dominant epi-allelic haplotypes (or epi-alleles) by grouping similar reads together to form chains of consecutive reads, called haplotype chains. These will vary in length depending on read length, sequencing depth, and data quality. In addition, the method provides information about genomic and epigenomic variation in the form of

- CpG methylation status
- Nucleosome occupancy (with NOMe-seq data)
- SNPs and SNVs

We use a read mapper (BSMAP [24]) to map reads to the reference genome (hg19). The performance of epiG depends on a number of user-adjustable parameters. These parameters control how noise is treated and how reads are clustered together to form haplotype chains. epiG starts by assigning each read to its own haplotype chain and then, iteratively, it improves the haplotype chains by re-assigning reads to chains one at a time, using an optimization procedure and prior genetic information. Each haplotype chain is characterized by an inferred DNA sequence annotated with CpG methylation and SNPs/SNVs. Noise and low frequency haplotype chains might further be filtered out by removing haplotype chains formed by few reads.

**Fig. 2:**
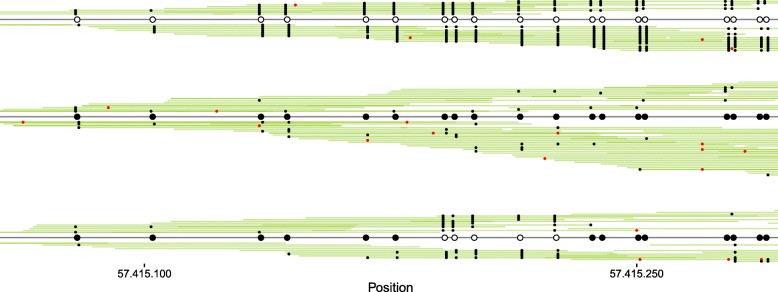
Example of haplotype chains. Illustration of the inferred haplotype chains in a 250-bp region (position 57,415,050–57,415,300 on chromosome 20) in the GNAS locus from colon tumor B (see ‘Methods’). One major and two minor chains are inferred. The major chain is fully methylated while one of the minor chains is completely unmethylated. Each line is one read (*green*), with paired reads on the same line. Converted CpGs are marked in *black*. Bases not matching the reference genome are marked in *red* and are indicative of sequencing errors. The reads in the top part of a chain have an inferred forward direction, while the reads in the bottom part have an inferred reverse direction. After assigning reads to haplotypes chains, the epigenotype of a chain is inferred from all reads in the chain. The epigenotype is shown in the middle of each chain. An *open circle* is an unmethylated CpG site and a *closed circle* is an methylated CpG site. The chains continue beyond the right end point, but are cut for illustrative purposes

Figure 2 shows an example of three distinct haplotype chains inferred from a region on chromosome 20 from a colon tumor sample (see ‘Methods’). One chain is completely unmethylated, while another chain is fully methylated. The last chain is partly methylated. Compared to a consensus method that outputs the average degree of methylation across all reads (irrespective of their epi-allelic haplotype), our method provides information about linked methylated sites on the same epi-allele as well as epi-allelic diversity.

In the following, we applied epiG to publicly available WGBS data sets and to two NOMe-seq data sets from our own lab. See ‘Methods’ and Table 2 for naming of and a description of the data sets. For all data sets, epiG default parameters were used. See ‘Methods,’ Table 3, and Additional file 1 for details.

**Table 2:** WGBS and NOMe-seq data sets

				Mapped	Read/		
Sample name	Description	Method	Layout	reads	paired length	GEO	Ref
WA9	Human embryonic stem cell line	WGBS	Paired	1360M	100/186	GSM1521762	[25]
Colon normal A	Sigmoid colon tissue	WGBS	Single	1703M	101/ –	GSM983645	[26]
Colon normal B	Primary colon adjacent to tumor tissue	WGBS	Paired	789M	100/191	GSM1204466	[15]
Colon tumor B	Colon primary adenocarcinoma	WGBS	Paired	807M	100/191	GSM1204465	[15]
PrEC	Prostate epithelial cells	NOMe-seq	Paired	293M	73/128	GSE94361	–
LNCaP	Prostate adenocarcinoma cell line	NOMe-seq	Paired	329M	100/146	GSE94361	–

In the text, we refer to the data sets by their sample names. The data sets colon normal A, PrEC, and LNCaP have lower data quality than the other data sets. The read/paired length is approximate. See Additional file 1: Figure S28 for the position-wise read depth for all samples

*WGBS* whole-genome bisulfite sequencing

**Table 3:** Default parameter values for WGBS and NOMe-seq data

	WGBS	WGBS	
Parameter	single	paired	NOMe-seq
*α*	0.95	0.95	0.95
*β*	0.05	0.05	0.05
*K*_0_	40	50	40
*K*_1_	1	2	0
*K*_2_	0	0	0
*K*_3_	0	0	2
*q*	0.9999	0.9999	0.9999

See also Additional file 1: Table S1. *K*_0_ and *K*_1_ are put to different values for single and paired reads, because paired reads are generally longer than single reads (see Table 2). The default values for *α* and *β* are conservative estimates of the failed bisulfite conversion rate and the inappropriate bisulfite conversion rate, respectively [32, 36]

*WGBS* whole-genome bisulfite sequencing

### Allele-specific methylation

To illustrate the performance of epiG to detect ASM, we ran epiG on the four WGBS data sets (WA9, colon normal A, colon normal B, and colon tumor B; see Table 2 and ‘Methods’) near genomic regions that are known to be allele-specific methylated. None of these data sets were used for this or similar purposes in the original publications [15, 25, 26]. We focused particularly on two well-described ASM regions, namely the GNAS locus on chromosome 20 and the *H19* non-coding gene on chromosome 7, both of which are known imprinted regions [9].

**Fig. 3:**
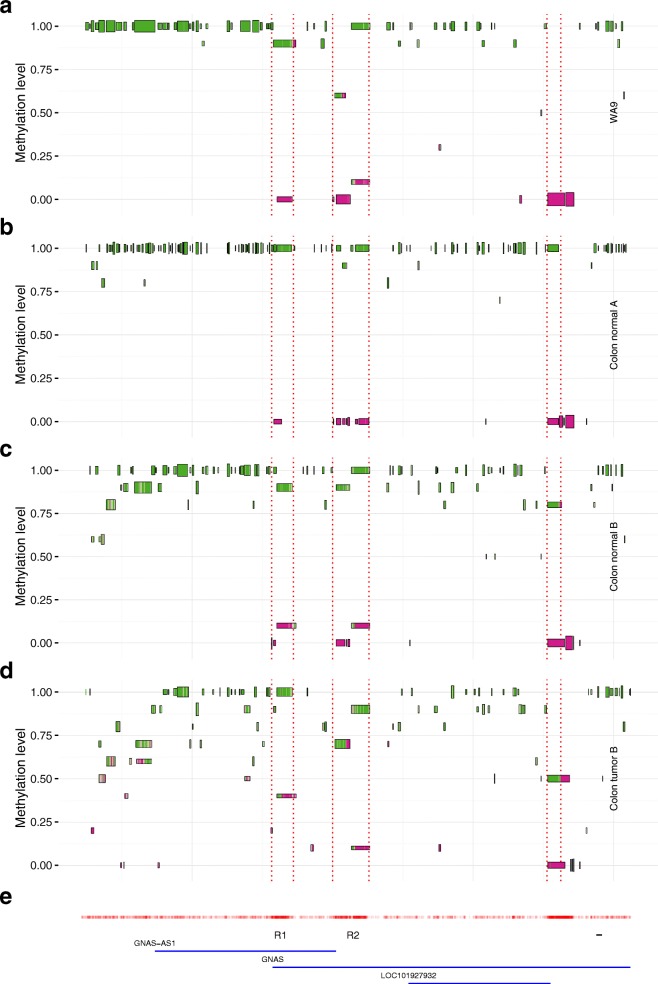
(See figure on previous page.) Epi-allelic profiling near the GNAS locus. Epi-allelic haplotypes of four samples in a 100-kb region near the GNAS locus with three known regions of ASM (R1, R2, and R3; *vertical dashed lines* [9]). **a** WA9. **b** Colon normal A. **c** Colon normal B. **d** Colon tumor B. **e** CpG site density map (*red*) and known genes (*blue*). In each of **a**–**d**, the *horizontal bars* are inferred epi-allelic haplotypes and the bar thickness represents the average number of reads per position. The degree of methylation is shown in two ways. Firstly, the average methylation level of three consecutive CpGs in a chain is shown in color, where *green* is for fully methylated CpG sites, *purple* for fully unmethylated, and *white* for neither. The haplotype overall methylation level is the average methylation level of all CpGs in the whole chain. In all three regions, we clearly see ASM. The stem cell sample (WA9) shows only one (unmethylated) epi-allele in R3. To the immediate right of R3, all four samples show consistently one unmethylated epi-allele. WA9 and colon normal A are fully methylated outside the ASM regions, whereas a general loss of methylation can be seen in colon tumor B and partly in colon normal B, which is tissue adjacent to the tumor. The density of CpGs is an important determinant of the length of the haplotypes. When the density is low, haplotypes become shorter and more fragmented. *ASM* allele-specific methylation

In the ASM regions near the GNAS locus, we find either fully methylated or fully unmethylated epi-alleles, strongly suggesting that there are two dominating epi-allelic haplotypes (see Fig. 3).

**Fig. 4:**
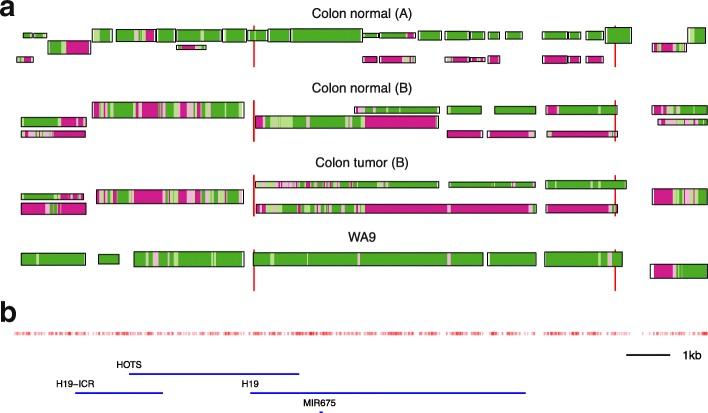
Epi-allelic profiling near the *H19* gene. Epi-allelic haplotypes of the four samples in a 20-kb region around the *H19* gene with one known ASM region (*vertical red lines* [9]). **a** *Horizontal bars* are inferred haplotype chains and the bar thickness represents the average number of reads per position. The average methylation level of three consecutive CpGs in a chain is shown in color, where *green* is for fully methylated CpG sites, *purple* for fully unmethylated, and *white* for neither (the vertical position of a chain represents the average degree of methylation). WA9 has only one (methylated) epi-allele in the entire ASM region, while the other three show one methylated and one unmethylated epi-allele. The unmethylated region does not span the entire ASM region, which is compiled from 22 methylomes from cell lines and tissue samples (not including the colon) [9]. **b** CpG site density map (*red*) and known genes (*blue*). The region ∼2–4 kb upstream of *H19* is the *H19* imprinting control region (*H19*-ICR). *ASM* allele-specific methylation

The paternal copy of the *H19* gene is usually methylated and silent while the maternal copy is hypomethylated or unmethylated [27]. We reach the same conclusion from three of the four data sets, except for the WA9 sample, which shows consistent methylation and a single epi-allelic haplotype throughout the region (see Fig. 4). This observation is in accordance with the previously described timing of *H19* gene activation to the peri-implantation stage of human embryo development [28]; thus, this illustrates the applicability of epiG in the study of ASM during embryonic development and cancer.

The region ∼2–4 kb upstream of *H19* is the imprinted control region *H19*-ICR, which is required to establish parent-of-origin imprinting of *H19* [29]. Essentially, we find a single epi-allelic haplotype in this region for each sample. It is methylated in WA9, but has a mixed methylation pattern in the three colon samples with chunks of unmethylated CpGs. If methylated, as in WA9, the upstream *IGF2* gene is activated and *H19* expression is silenced [29].

Figure 5 shows summary statistics for different genomic regions in the four different samples. For allele-specific methylated regions (AMRs), we generally find one or two distinct haplotype chains, while for other regions (promotor, exon, and randomly selected regions), we generally find one haplotype chain, indicating that both DNA copies of a region are methylated in the same way. Additional file 1: Figures S1–S18 show that the number and extent/length of haplotype chains in 18 AMRs [9] in the four samples generally are in high agreement. The position-wise variance of the number of haplotype chains is as low as 0.22 across all positions and AMRs. In contrast, randomly assigning one or two chains to each region would yield a variance of 1.

**Fig. 5:**
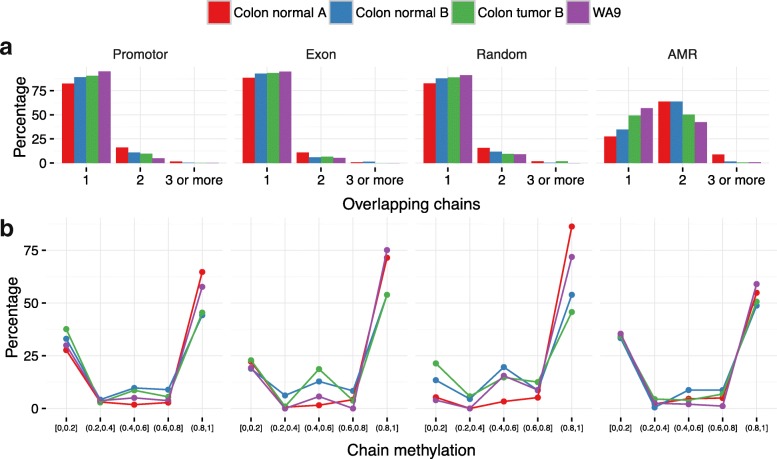
Summary statistics of the estimated haplotype chains. Collected statistics of the estimated haplotype chains over four different genomic regions: promoters, exons, randomly selected regions, and known AMRs. For each type, except AMR, 100 regions where randomly chosen. For AMR, 18 known AMRs were used. See Additional file 1: Table S2 and [9]. For each region, haplotypes were estimated using the default configuration and the noise was reduced as described in ‘Noise reduction’ in ‘Results and discussion.’ **a** The percentage of regions with only one chain, two overlapping chains, and three or more overlapping chains for each region. The majority of regions, except AMRs, were covered by only one chain. **b** The distribution of the average methylation level over the haplotype chains. Chains tend to be either fully methylated or completely unmethylated. *AMR* allele-specific methylated region

### Nucleosome occupancy

The NOMe-seq protocol was developed for simultaneously assessing nucleosome positioning and DNA methylation genome-wide [18]. This protocol uses the ability of the M.CviPI methyltransferase enzyme to methylate the cytosine in a GpC dinucleotide in accessible chromatin. Thus, M.CviPI treatment of intact cell nuclei can demarcate the nucleosome positioning of cells. Following bisulfite conversion and whole-genome sequencing, it is, therefore, possible to infer nucleosome positioning using artificial GpC-methylation as well as endogenous DNA methylation at CpG dinucleotides on the same molecule [18].

Only isolated GpC sites (*DGCH* sites, or sites not bordering C to the left and G to the right; see ‘Notation and definitions’ in ‘Methods’) can be used for nucleosome inference, as otherwise the signal might be confused with endogenous CpG methylation [18].

**Fig. 6:**
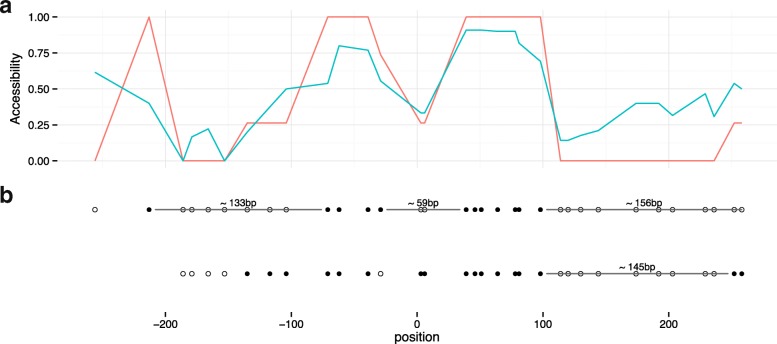
DNA accessibility near a CTCF site. **a** Consensus DNA accessibility near a representative CTCF site at position 31,109,691 on chromosome 20 in the LNCaP sample. The center of the CTCF site is at position 0 of the plot. *Red line*: consensus based on the two chains shown in (**b**); *blue line*: consensus based on all reads. **b** The two dominating epi-alleles. Each point is an isolated GpC position. *Black*: methylated GpC position (DNA accessible), *white*: unmethylated GpC position (DNA inaccessible). The approximate lengths of the inaccessible intervals are computed (*black lines*), showing that the CTCF site appears to be flanked by nucleosomes (∼149 bp). In one epi-allele, the CTCF site appears to be inaccessible, potentially due to binding of a protein. As nucleosome positioning is dynamic and the read coverage is low for these samples, the inferred intervals are uncertain

**Fig. 7:**
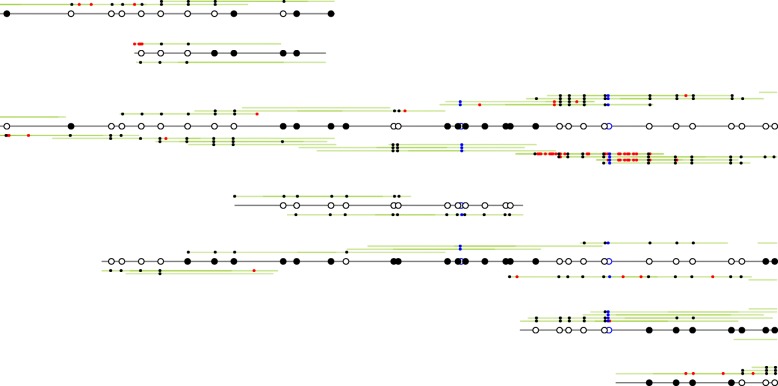
Haplotype chains near a CTCF site. The inferred haplotype chains in a small 500-bp region near the CTCF site at position 31,109,691 on chromosome 20 (same as in Fig. 6) in the LNCaP sample. Seven chains are found (*black lines with open and closed circles*). The two major chains are also shown in Fig. 6. The reads (*green lines*) assigned to a haplotype chain are shown above (inferred forward direction) and below (inferred reverse direction) the chain, with paired reads on the same line. Converted isolated CpGs are marked in *blue* and converted isolated GpCs in *black*. Bases not matching the reference genome are marked in *red* and indicative of errors (sequencing or bisulfite conversion). After assigning reads to haplotypes chains, the epigenotype of the chain is inferred from the reads: converted GpCs (*solid black circles*), unconverted GpCs (*open black*), and converted CpGs (*open blue*; there are only two of these)

We applied epiG to NOMe-seq data from the PrEC and LNCaP samples from our own lab (see Table 2 and ‘Methods’). CTCF binding sites have been shown to have characteristic DNA methylation and nucleosome occupancy patterns. Figures 6 and 7 show an example of nucleosome inference near a CTCF site where different epi-allelic haplotypes can be distinguished. In some of these, the CTCF site is inaccessible due to protein binding. As nucleosome positioning is dynamic and varies across cells, inference on nucleosome length and position is uncertain except in regions where stable nucleosomes are expected [30, 31]. When averaging over many CTCF sites, we see a clear periodic pattern, like what is reported in [18] (see Additional file 1: Figure S19).

### Noise reduction

We are mainly interested in the dominant epi-alleles of a sample. Therefore, we filter out minor epi-alleles and reduce noise by excluding haplotype chains with few reads or low read coverage per position. This is a post-inference step that affects the number of reported haplotype chains and how many haplotypes are filtered out. Thus, the number and the form of the dominating haplotype chains (as those in Figs. 3 and 4) are unaffected by this filtering step.

The depth fraction of a chain is defined as

$$\frac{\text{Total base pairs in chain}}{\text{Total base pairs overlapping the chain}}, $$

where the total base pairs in chain is the sum of the lengths of all reads in the chain and the total base pairs overlapping the chain is the sum of the lengths of all reads in the chains overlapping the particular chain.

**Fig. 8:**
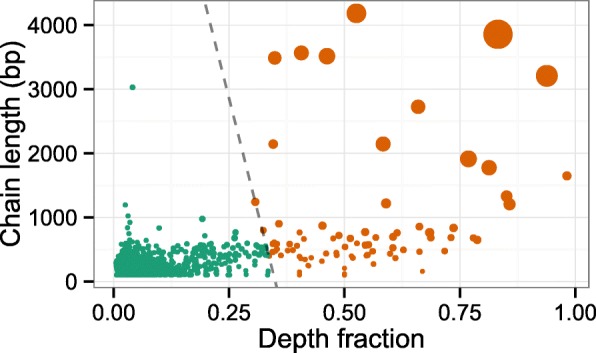
Depth fraction and length of haplotype chains. Plot showing the lengths of haplotype chains against the depth fraction of the chains in a 100-kbp region near the GNAS locus for the WA9 sample. Each *point* is a chain and the size indicates the number of reads within it. To reduce noise and to extract the dominating haplotype chains, chains falling to the left of the *straight line* are removed as they either have a short length or contain a low fraction of the reads in the region. *Green*: removed chains. *Brown*: kept chains. The exact same cut-off was applied to all samples in all analyses. The chains are plotted against the genomic position in Fig. 9 and in Fig. 3 after the removal of noise

**Fig. 9:**
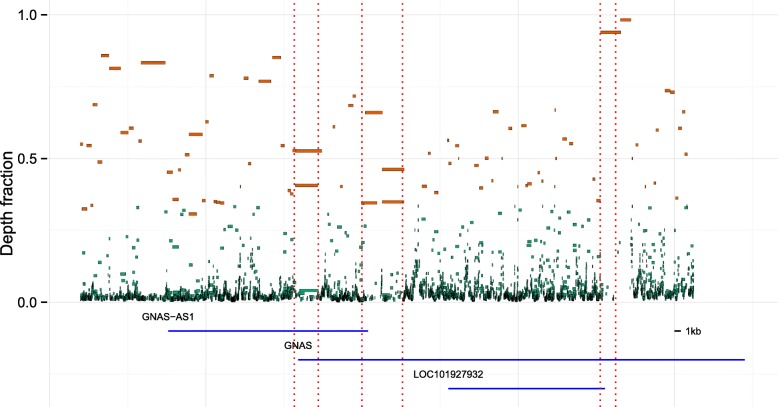
Inferred haplotype chains. The plot shows all inferred haplotype chains in a 100-kb region near the GNAS locus in the WA9 sample before noise reduction, as Fig. 3. The depth fraction of a chain is plotted against its genomic position. The color indicates whether the chain is kept (*brown*) or interpreted as noise (*green*). See Fig. 8

We use the depth fraction to distinguish noise from signal and do so by only keeping chains for which the inequality

$$a \le \text{chain length} + b\times\text{depth fraction} $$

holds, for some values of *a* and *b*. In general, this means that we keep long chains and chains with a high depth fraction. In all presented analyses, we used *a*=10×10^3^ and *b*=28.5×10^3^ for paired layout WGBS, and *a*=5×10^3^ and *b*=20×10^3^ for single layout WGBS, based on empirical observations (see Fig. 8, Fig. 9, and Additional file 1: Figures S20, S21, and S22). With the chosen values, the main determinant of whether a chain is kept or not is the depth fraction (see the figures). As seen in the figures, a common feature is a trail of haplotype chains with very low depth fraction, corresponding to haplotype chains consisting of very few reads. There is not a gold standard for setting *a* and *b*.

### Validation and benchmarking

In addition to the qualitative validation presented above, we carried out quantitative validations. Specifically, we measured the ability of epiG to infer position-wise methylation and SNP genotypes by comparing epiG predictions to measurements obtained from Infinium BeadChip and SNP6.0 arrays, with details given below. The results were also compared to results obtained using Bis-SNP [20], a state-of-the-art method for inferring position-wise methylation and genotypes from WGBS and NOMe-seq data. Further, we investigated the robustness of epiG by varying the default parameters.

#### Methylation validation

To evaluate the inferred methylation states, we used ∼18k isolated CpG sites (*HCGD* sites; see ‘Notation and definitions’ in ‘Methods’) with high and low methylation levels on the Infinium BeadChip from the LNCaP sample. We used isolated sites to distinguish endogenous methylation from methylation caused by the GpC methyltransferase [18].

We first preselected the 5% of CpG sites with the highest *β* value (high) and the 5% with the lowest *β* value (low) on the Infinium BeadChip from the LNCaP sample (see ‘Methods’ for details). Subsequently, we removed all non-isolated CpG sites and ended up with approximately 18k isolated CpG sites for validation of which ∼8k have low *β* values and ∼10k have high *β* values. We took high and low as being the true methylation states of the sites. For each of the 18k CpG sites, we then predicted the states high and low from the methylation levels inferred by epiG and Bis-SNP.

For epiG, the inferred level is the percentage of reads with inferred CpG methylation. Bis-SNP outputs automatically an inferred percentage for each site. We calculated the receiver operating characteristic (ROC) curves for epiG and Bis-SNP (see Additional file 1: Figure S23), and chose the thresholds that gave the best classifiers for epiG and Bis-SNP. Specifically, we chose the thresholds that minimized the squared sum FP^2^+(1−TP)^2^, where FP is the false positive rate and TP the true positive rate. The inference failed for 1% (10%) of the sites for epiG (Bis-SNP). For epiG, these are sites not covered by any reads or sites for which the sequenced nucleotide is uncertain (N).

**Fig. 10:**
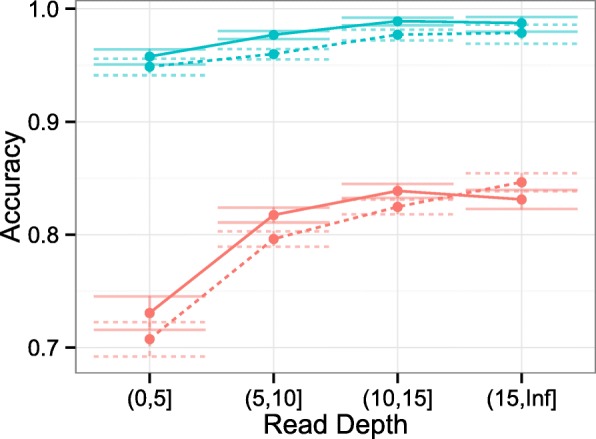
Position-wise methylation and SNP validation. Accuracy plotted against read depth for epiG (*solid line*) and Bis-SNP (*dotted line*). *Blue lines* are CpG consensus methylation predictions and *red lines* SNP predictions. 95% confidence intervals are shown. The two methods show comparable results. *SNP* single nucleotide polymorphism

Using the optimized thresholds, in Fig. 10 we plotted the fraction of true predictions against the read depth. Not surprisingly, and for both methods, the accuracy increases with read depth and was above 90% when the read depth is higher than five reads.

#### Genotype validation

For SNP genotype validation, we selected all high-confidence genotype calls (∼74k) on chromosomes 1 and 2 on the SNP6.0 array from the LNCaP sample (see ‘Methods’). The genotype calls on the array were taken to be the true genotypes. Of the ∼74k SNPs, 170 had zero read depth (no reads available) and, hence, inference failed for both epiG and Bis-SNP for these sites.

For epiG, the combined genotype is computed from the haplotype chains with log-likelihood ratios *F*≤−15 or |*F*_*R*_−*F*_*A*_|≥15 (see ‘Log-likelihood ratios’ in ‘Methods’ for definitions). This is done to avoid haplotype chains with very few reads affecting the combined genotype. The combined genotype is then the genotype represented by the remaining haplotype chains. For example, if there are two remaining haplotype chains with genotype C and one with T then the combined genotype is *CT*.

The overall result of the genotype validation can be seen in Fig. 10. The performance differs for the different SNP genotypes (see Additional file 1: Figure S24). It is best for the homozygous genotypes and less good for the genotypes *AG* and *CT*, where confusion with methylated cytosines is possible.

#### Robustness

epiG depends on a number of user-adjustable parameters. To investigate the robustness of the performance of epiG, we varied the default parameters and compared the results for different parameter choices. Of particular interest are the following questions: (1) How does the methylation and genotype validation depend on the parameters? (2) How does the number of dominating haplotype chains vary with the parameters? Different choices of parameters might divide haplotype chains into several smaller chains or create fewer longer chains by merging smaller chains. An important issue is whether the number of chains overlapping a particular site in the genome remains the same for different parameter values. Refer to ‘Methods’ and Table 3 for a description of the parameters.

Additional file 1: Figure S25 shows the results of varying the failed conversion rate *α* and the inappropriate conversion rate *β* on the methylation calls. Specifically, we calculated the area under the curve (AUC) of the ROC curve, as in Additional file 1: Figure S23, for a grid of *α* and *β* values (including the default values). The AUC changes only by less than 2%, yielding high robustness in calling methylation status.

Additional file 1: Figure S26 shows the influence of the genotype prior on the genotype calls. The prior depends on a single adjustable parameter *q* and uses information from hg19 and dbSNP135 (optional), weighted by *q*; see ‘Methods’ for details. If dbSNP135 is not used, then epiG assumes there is only one possible genotype for each genomic position, except for private mutations. We repeated the genotype validation study on the same ∼74k SNPs using different configurations of the prior. The percentage of correctly called *AA* and *TT* genotypes is unaffected by the prior settings (including with and without dbSNP135). For the genotypes *CC* and *GG*, the situation is different. Due to the bisulfite conversion process, the true genotypes might be confused with the genotypes *CT* and *GA*, respectively, if dbSNP135 is used. In fact, if the read depth is *high*, we see a marked decline in the performance, mainly for low *q*. This is not the case if dbSNP135 is not used. The calls of the heterozygous genotypes are also affected by changes in the prior setting, again because the bisulfite conversion process creates ambiguities in the interpretation of the data. However, in this case dbSNP135 helps resolve ambiguities unlike for the *CC* and *GG* genotypes. The percentage of correctly called SNPs increases by up to ∼30 percentage points with the inclusion of dbSNP135, depending on SNP type and read depth. Based on these investigations, we advocate the use of dbSNP135 and a high *q*.

Note that these results might be extrapolated to non-SNP positions as this corresponds to having a homogeneous genotype and not using dbSNP135. From the discussion above, we conclude that epiG performs well for non-SNP positions. The performance for private mutations is not clear and could be negatively influenced by the prior.

Additional file 1: Figure S27 shows that the number of haplotype chains overlapping a particular site in the genome essentially remains unchanged when varying the values of *K*_0_≥10 (bp) and *K*_1_≥1 (CpG sites), which control the required overlap between reads and haplotype chains. This is, in particular, the case for the ASM regions. However, individual haplotype chains might still be split up or merged according to the values of *K*_0_ and *K*_1_.

## Conclusions

Various statistical methods exist for drawing inference on epigenetic patterns in general and ASM specifically. Admixture models, e.g. as implemented in armfinder [9], have been used to identify AMRs from WGBS data without inferring the structure of the underlying epi-allelic haplotypes. Other methods, such as Bis-SNP [20] and Bismark [21], infer CpG methylation levels position-wise (and they output the degree of methylation) from WGBS data without distinguishing between epi-alleles.

We have developed a novel method to infer CpG methylation states from WGBS data and epigenetic states, including DNA methylation and nucleosome positioning, from NOMe-seq data. The method groups similar reads into haplotype chains, thereby making it possible to draw inference at the haplotype level rather than at the nucleotide level alone. In particular, we have demonstrated that epiG is able to infer allele-specific methylated epi-alleles in different WGBS samples and to reveal information about nucleosome occupancy in NOMe-seq data. The separation of reads into distinct epi-allelic haplogroups is a first step in providing a statistical method that is able to extract information about epigenetic mixtures of cell populations and their frequencies, and ultimately enable the comparison of epigenetic states between cell types.

We benchmarked epiG against array data from prostate cells grown in culture and showed that epiG performed comparably to Bis-SNP with respect to position-wise methylation. The software epiG is available for download (see ‘Software’ in ‘Methods’ for details).

## Methods

### Statistical model

**Fig. 11:**
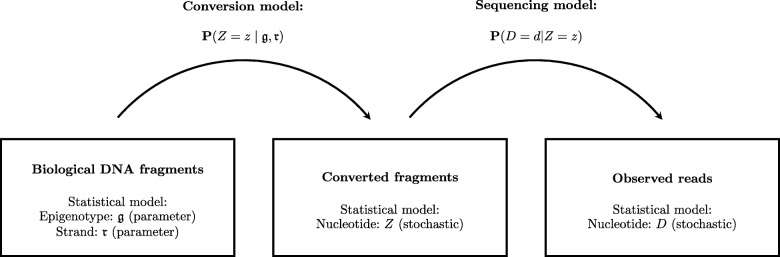
Outline of the statistical model

We first formulate a statistical model for WGBS (and NOMe-seq) data based on the bisulfite sequencing protocol described in [17]. The model takes into account sequencing errors, and failed and inappropriate bisulfite conversions [32], as well as errors in methylation of GpC sites (NOMe-seq data only) [18]. The model consists of a conversion model and a sequencing model. See Fig. 11 for the overall structure.

The true *epigenotype* of a position in a DNA fragment is one of

$$ \mathsf{C},\mathsf{G},\mathsf{A},\mathsf{T}, \overset{\text{me}}{\mathsf{C}}, \,\,\text{or}\,\, \underset{\text{me}}{\mathsf{G}}, $$

representing the nucleotides cytosine, guanine, adenine, and thymine, plus methylated cytosine and guanine complementary to a methylated cytosine on the reverse strand, respectively. (The epigenotype is haploid and not diploid as is normally the case.) We observe the epigenotype subject to two levels of noise: first of all, the DNA fragment is potentially treated with GpC methyltransferase (NOMe-seq), then bisulfite converted and PCR amplified. Secondly, it is passed through a sequencing machine. For NOMe-seq data, we take the methylation state of the enzyme-treated genome to be the true epigenotype.

We are interested in the probability of the observed nucleotide *D* in a given position in a read as a function of the epigenotype *g* and the strand *r* (fwd,rev) from which the read originates. Both *g* and *r* are in this context unknown parameters that we aim to estimate. The probability is modeled in two steps, reflecting the two levels of noise.

First, we let *Z* be the nucleotide at the position in the DNA fragment after conversion and PCR amplification. The probability of *Z* depends on the parameters *g* and *r*, the rate *α* of failed bisulfite conversions, and the rate *β* of inappropriate bisulfite conversions. It is given in Table 4 and builds on the observations in Fig. 1.

**Table 4:** The bisulfite conversion model

	Epigenotype *g*					
*Z*	C	G	A	T	$\overset {\text {me}}{\mathsf {C}}$	$\underset {\text {me}}{\mathsf {G}}$
C	$\hat \rho \alpha $	0	0	0	$1 - \hat \rho \beta $	0
G	0	*ρ* *α*	0	0	0	1−*ρ* *β*
A	0	1−*ρ* *α*	1	0	0	*ρ* *β*
T	$1 - \hat \rho \alpha $	0	0	1	$\hat \rho \beta $	0

The probability **P**(*Z*=*z*∣*g*,*r*) of *Z* given the epigenotype *g* and the strand *r*. Here *α* is the rate of failed bisulfite conversion and *β* is the rate of inappropriately converted nucleotides. The parameter *ρ* is 0 if *r*=fwd, 1 if *r*=rev, and $\hat \rho =1-\rho $. For example, **P**(*Z*=C∣*g*=C,*r*=fwd)=*α*

Second, the probability of *D* given *Z* depends on the reliability of the base-calling. We treat the reliability as an additional parameter, *ε*, in the model and obtain this parameter from the Phred sequencing quality scores [33, 34]. Specifically, we put

$$ \mathbf{P}(D = d \mid Z = z) = \left\{ \begin{array}{lll} 1-\epsilon, & \text{if} & d = z, \\ \frac{\epsilon}{3}, & \text{if} &d \neq z, \end{array}\right. $$

for *d*,*z*=C,G,A,T. As we treat N as an unknown nucleotide, we have **P**(*D*=N∣*Z*=*z*)=1. We have

1 $$\begin{array}{@{}rcl@{}} \lefteqn{\mathbf{P}(D=d\mid g,r)}  \\ & = & \sum_{z\in\{\mathsf{C},\mathsf{G},\mathsf{A},\mathsf{T}\}}\mathbf{P}(D = d \mid Z = z)\mathbf{P}(Z = z\mid g,r)  \\ &= & \left(1-\frac{4}{3}\epsilon\right)\mathbf{P}(Z=d\mid g,r) + \frac{\epsilon}{3}, \end{array} $$

for *d*=C,G,A,T and **P**(*D*=N∣*g*,*r*)=1.

The model can be made position or context dependent by changing the default settings (see Additional file 1). In particular, the quality score always depends on the position.

### Overview of the inference procedure

The sequenced reads come from different cells that might or might not share epigenetic structure. Typically, we find several non-compatible epigenetic haplotypes, as well as reads that support a common, or similar, origin. We call a collection of DNA fragments from the same epigenetic haplotype for an *epigenetic haplotype chain* or just a haplotype chain.

We propose an algorithm that does the following:

1. Infer haplotype chains, that is, cluster the reads according to their epigenetic haplotype.
2. Infer the epigenotype of each position and strand of each read in a haplotype chain.

These inference steps are done using a constrained maximum posterior procedure based on the model Eq. 1, as described in the ‘Details of the inference procedure.’

#### Notation and definitions

We use standard nomenclature and let H be any nucleotide different from G, and D be any nucleotide different from C [35]. A CpG site is said to be isolated if the context is *HCGD* and likewise, a GpC is said to be isolated if the context is *DGCH*.

Let *n* denote the number of reads and *b*_*i*_ the haplotype chain of read *i*. If read *i* and *j* come from DNA fragments with the same epigenetic haplotype, then *b*_*i*_=*b*_*j*_. The strand of read *i* is called *r*_*i*_. We refer to *B*=(*b*_1_,…,*b*_*n*_) as the *haplotype structure* and to *R*=(*r*_1_,…,*r*_*n*_) as a *strand assignment*.

Each position in a haplotype chain has a unique epigenotype. For a haplotype chain *b*, let

2 $$\begin{array}{*{20}l} g_{b} =\left(g_{S_{b} b},\dots, g_{\left(S_{b}+L_{b}-1\right)b}\right) \end{array} $$

be an assignment of an epigenotype to each position in the chain, where *S*_*b*_ is the start position of the chain (with respect to a reference genome) and *L*_*b*_ the length of the chain. We refer to *g*_*b*_ as an *epigenotype chain*. The collection of epigenotype chains, one for each haplotype chain, is denoted by *G* and referred to as an *epigenotype assignment*.

An epigenotype chain is said to be *strand compatible*, if the epigenotypes of each CpG site are either *CG* or $\overset {\text {me}}{\mathsf {C}}\underset {\text {me}}{\mathsf {G}}$, that is, if C is methylated on the forward strand, then the C on the reverse strand complementary to G is also methylated. In NOMe-seq mode, this is required only for isolated CpG sites as well as all isolated GpC sites, for which the epigenotypes should be *GC* or $\underset {\text {me}}{\mathsf {G}}\overset {\text {me}}{\mathsf {C}}$.

Let $\overline {g}$ denote the nucleotide of an epigenotype *g*. For example, if *g*=C, then also $\overline {g}=\mathsf {C}$ and if $g=\overset {\text {me}}{\mathsf {C}}$, then $\overline {g} = \mathsf {C}$.

#### Feasible haplotype chains

For a haplotype chain *b*, let *c*(*b*,*j*) be the read depth of the chain at position *j*, that is, *c*(*b*,*j*)≥0 is the number of reads in the chain overlapping position *j*. The range of the haplotype is the interval [*S*_*b*_,*S*_*b*_+*L*_*b*_−1] from the start position of the first read of the chain until the end position of the last read of the chain.

A haplotype chain *b* with epigenotype chain *g*_*b*_ is said to be *feasible* if

1. It has positive read depth throughout its range, that is, if *c*(*b*,*j*)>0 for all *j*∈ [ *S*_*b*_,*S*_*b*_+*L*_*b*_−1].
2. It consists of a single read, or it is obtained from another feasible haplotype chain *b* ^′^ by adding one read *i*_*b*_, such that
  - The overlap between *b* ^′^ and *i*_*b*_ is at least *K*_0_ positions.
  - The overlap between *b* ^′^ and *i*_*b*_ contains at least *K*_1_ CpGs, *K*_2_ isolated CpGs, and *K*_3_ isolated GpCs.
3. The epigenotype chain *g*_*b*_ is strand compatible.

A haplotype structure *B* is said to be feasible if all haplotype chains of *B* are feasible. The haplotype structure that assigns every read to its own haplotype chain is feasible.

Default values for *K*_0_,*K*_1_,*K*_2_, and *K*_3_ are listed in Table 3. A high overlap between two reads makes it more likely the reads originate from the same epi-allelic haplotype, than a small overlap. However, if we require a large overlap, few reads will cluster together, leading to many haplotype chains with few reads. There is, therefore, a balance to strike. If reads are long, we can in general ask for a larger overlap than if reads are small. If two reads do not overlap in any CpGs, then there is no epigenetic evidence they are from the same epi-allelic haplotype. Hence *K*_1_ should be at least 1.

### Details of the inference procedure

#### The likelihood and priors

The haplotype structure *B*, the strand *R*, and epigenotype assignment *G* are unknown parameters and will be inferred from the data. The parameters *α*,*β*, and *ε* will be fixed (assumed known) in the inference procedure and not estimated from the data.

The lengths of the reads are denoted by *l*_1_,…,*l*_*n*_. We observe the nucleotide sequences

$$\left(d_{i1}, \dots, d_{{il}_{i}}\right) \in \left\{\mathsf{C},\mathsf{G},\mathsf{A},\mathsf{T},\mathsf{N}\right\}^{l_{i}}, \quad i = 1, \dots, n, $$

of the *n* reads. In our data analyses, *l*_*i*_ is typically around 75–100.

With this in mind, the likelihood of the observed reads is given by

$$\mathcal{L}(B, G, R) = \prod_{i=1}^{n}\prod_{j=1}^{l_{i}} \mathbf{P}(D = d_{ij}\mid g_{(s_{i}+j)b_{i}}, r_{i}), $$

where *s*_1_,…,*s*_*n*_ are the start positions of the reads in the genome. Here $g_{(s_{i}+j)b_{i}}$ is the epigenotype at the *j*th position in the *i*th read of the haplotype chain $g_{b_{i}}$ containing the read (see Eq. 2). The parameters *α*, *β*, and *ε* are suppressed in **P** as they are not estimated from the data.

We add priors on *B*,*G*, and *R*. We use a uniform prior on *R*, such that all strand assignments a priori are equally likely. The prior on *B* controls the number of haplotype chains, that is, the tendency of reads to be grouped into the same haplotype chain (potentially at the cost of imposing errors). The prior on *G* is derived from hg19 (reference genome) and dbSNP135. The purpose of this prior is to guide the inference in situations of low read depth, abundant errors, or high methylation levels.

#### Optimization

The posterior likelihood for the optimization problem is defined as

3 $$ \Lambda(B, G, R)= \pi_{0}(B) \pi_{1}(G\, | B)\mathcal{L}(B,G,R),  $$

where *π*_0_(*B*) and *π*_1_(*G* |*B*) are the priors on the haplotype structure and the epigenotype assignment, respectively. As the prior on *R* is uniform, it can be omitted. In other words, *Λ*(*B*,*G*,*R*) is the likelihood of the data weighted by *π*_0_(*B*) and *π*_1_(*G* |*B*).

The inference procedure updates estimates of *B*,*G*, and *R* iteratively, such that the posterior likelihood increases in each step. In the (*k*+1)th step, new estimates $\widehat B_{k+1}$ and $\widehat R_{k+1}$ are proposed from the estimates $\widehat B_{k}$ and $\widehat R_{k}$ in the previous step. Given $\widehat B_{k+1}$ and $\widehat R_{k+1}$, the optimal estimate $\widehat G_{k+1}$ of *G* can be computed directly (see Additional file 1). The algorithm is guaranteed to converge in a finite number of steps. However, in rare cases, the final estimates are found to be suboptimal (see Additional file 1). The initial haplotype structure assigns all reads to their own haplotype chain. Note that if we do not use a prior on *B*, then the algorithm will remain in the initial configuration, as the likelihood does not increase by re-assigning reads to other chains.

For further details and implementation, see Additional file 1.

#### Priors

Let *I*_*b*_ denote the set of all reads in haplotype chain *b*, hence *I*_*b*_={*i*=1,…,*n*∣*b*_*i*_=*b*}. The prior on *B* is defined as

$$\pi_{0}(B) \propto \prod_{i=1}^{n}\sqrt{L_{b_{i}}} \left(\sum_{j \in I_{b_{i}}} l_{j} \right)^{\!2}. $$

The prior gives higher weight to longer chains as well as to chains for which the total length of all reads is high. If all reads have the same length, then

$$\pi_{0}(B) \propto \prod_{i=1}^{n}n_{i}^{2} \sqrt{L_{b_{i}}}, $$

where *n*_*i*_ is the number of reads in $I_{b_{i}}$, and the prior simply weights the number of reads in the chains to their length. The exponents could be chosen in many ways. Here the number of reads in a chain weights higher (exponent 2) than the length of the chain (exponent 0.5); thus, we favor thick chains to long (thin) chains. This is sensible, as we are particularly interested in the dominating epi-alleles.

The general form of the prior is based on practical considerations. The space of all haplotype structures *B* is a highly complex space and for computational reasons is it essential that the prior weight is relatively straightforward to calculate.

We assume the prior *π*_1_(*G* |*B*) is of the form

$$\pi_{1}(G\, |B) \propto \prod_{j}\prod_{b \in B(j)}q^{\mathbb{I}\left(\overline{g}_{jb}\right)}(1-q)^{1-\mathbb{I}\left(\overline{g}_{jb}\right)}, $$

where the first product is over all positions in the haplotype chains, *B*(*j*) is the set of haplotype chains overlapping position *j*, $\overline {g}_{jb}$ is the nucleotide of position *j* in chain *b* (Eq. 2), and $\mathbb {I}(\overline {g}_{jb})$ is 1 if the genotype is found in hg19 or dbSNP135, and it is 0 otherwise (dbSNP135 is optional). If *q*=0.5, then *π*(*G* |*B*) is independent of hg19 and dbSNP135 and gives the same prior weight to all observations. The prior serves two purposes: the main purpose is to guide epiG in situations where the data might be interpreted in different ways because the bisulfite conversion process creates ambiguities; secondly, it weights the likelihood of introducing private mutations. We take *q*=0.9999 and use the same value of *q* for all data sets, even though it might be reasonable to adjust the parameter according to what is believed about the sample; for example, cancer cells are generally more exposed to private mutations than other cell types.

There is a missing normalization constant obtained by normalizing the distribution *π*_0_(*B*)*π*_1_(*G* |*B*) to 1. It has no influence on the estimation procedure. In practice, we, therefore, ignore the normalization constant.

#### Log-likelihood ratios

To assess the fitted epigenotypes, we compute three likelihood ratios. For a given haplotype structure and strand assignment, we compute a log-likelihood for each haplotype chain *b* that assesses the significance of a particular epigenotype *x* in position *j* in the chain,

$$\ell(x) = \sum_{b_{i}=b} \log\mathbf{P}(D = d_{ij}\mid x, r_{i}), $$

where the sum is over all reads in the chain overlapping the position (see Eq. 1). The dependence on *b* and *j* is suppressed in *ℓ*(*x*).

The log fit-ratio is defined as

4 $$ F=2\left(\max_{x \not\in \{g, \overline{g}\}}\ell(x)-\max_{x \in \{g, \overline{g}\}}\ell(x)\right),  $$

where *g* is the inferred epigenotype in the position. The log fit-ratio measures how well the inferred epigenotype (or the corresponding unmethylated nucleotide) fits compared to any other epigenotype. It can take any value since *ℓ*(*x*) is only maximized as part of the full posterior likelihood (see Eq. 3).

Additionally, the fit of the genomic reference nucleotide (or the alternative nucleotide, if relevant) can be similarly assessed:

5 $$ F_{R}=2\left(\max_{x \not\in \{y, y^{\text{me}}\}}\ell(x)-\max_{x\in \{y, y^{\text{me}}\}}\ell(x) \right)  $$

(similarly *F*_*A*_ for the alternative nucleotide), where *y* denotes the reference (or alternative) nucleotide and *y* ^me^ denotes the methylated nucleotide when *y*=C,G. Again, this quantity can take any value.

### WGBS data

We downloaded four publicly available WGBS data sets (see Table 2 and Additional file 1: Figure S28 for details). All data sets were mapped to the hg19 reference genome using BSMAP with the standard configuration [24]. For a paired design, reads not properly paired were removed. The bam file was sorted using Picard tools [37].

### LNCaP and PrEC cell culture

LNCaP prostate adenocarcinoma cells and PrEC normal primary prostate epithelial cells were obtained from the American Type Culture Collection (ATCC). LNCaP cells were grown in RPMI 1640 with L-glutamine supplemented with 10% fetal bovine serum, 100 U/ml penicillin, and 100 µg/ml streptomycin (Life Technologies). The authenticity of the LNCaP cell line was confirmed by short tandem repeat analysis (www.identicell.dk). PrEC cells were grown in Prostate Epithelial Cell Basal Medium (ATCC) supplemented with Prostate Epithelial Cell Growth Kit (ATCC). LNCaP and PrEC cells were harvested at 80% confluence using Trypsin-EDTA (Life Technologies) or Trypsin-EDTA for primary cells (ATCC) and Trypsin Neutralizing Solution (ATCC), respectively. For microarray analyses, genomic DNA from PrEC and LNCaP cells was extracted using the Puregene DNA purification kit (Gentra Systems) with proteinase K treatment (100 U, 30 min at 55 °C), as described previously [38].

### Microarray analyses

All microarray analyses were performed by service provider Aros Applied Biotechnology, Aarhus, Denmark. For SNP genotyping of LNCaP and PrEC cells, genomic DNA was labeled and hybridized to the Genome-Wide Human SNP6.0 array (Affymetrix, Santa Clara, CA, USA), as described previously [39]. SNP6.0 data processing and analysis were performed as previously [39].

For methylation profiling, 1 µg of DNA was bisulfite converted, whole-genome amplified, and analyzed on the Infinium Human Methylation450 BeadChip (Illumina, San Diego, CA) according to the protocol provided by the manufacturer. This array interrogates the methylation states of >485,000 CpG sites per sample at single nucleotide resolution, where each investigated CpG site is assigned a *β* value ranging from 0 (fully unmethylated) to 1 (fully methylated), corresponding to the ratio of the methylated signal divided by the sum of the methylated and unmethylated signals. Normalized peak-corrected *β* values were used.

### Genome-wide nucleosome footprinting assay

NOMe-seq was performed as previously described [18]. Briefly, exponentially growing cells were washed with phosphate buffered saline, trypsinized, and incubated with ice-cold lysis buffer (10 mM Tris, pH 7.4, 10 mM NaCl, 3 mM MgCl_2_, 0.1 mM EDTA, and 0.5% NP-40) for 5 min on ice to isolate intact nuclei. Nuclei were washed with ice-cold wash buffer (10 mM Tris, pH7.4, 10 mM NaCl, 3 mM MgCl_2_, 0.1 mM EDTA), resuspended in ice-cold 1 × GpC buffer (New England BioLabs), and treated with 200 units of M.CviPI enzyme supplemented with 1.5 µL S-adenosylmethionine (SAM) for 7.5 min with a boost of 100 units enzyme and 0.75 µL SAM for an additional 7.5 min. Genomic DNA was isolated by standard phenol-chloroform extraction and ethanol precipitation. WGBS libraries were generated using 2–5 µg of DNA as previously described and sequenced on a HiSeq 2000 performed by the USC Epigenome Center [40]. Sequencing reads were mapped to the hg19 reference genome using BSMAP.

## Additional file

Additional file 1 Additional information and figures. Additional information about the statistical method, in particular, the optimization and implementation of epiG, as well as additional figures referenced in the main text. (PDF 1880 kb)
