# Supplementary material for: epiG: statistical inference and profiling of DNA methylation from whole-genome bisulfite sequencing data
Source: Genome Biol. 2017 Feb 21;18:38. doi: 10.1186/s13059-017-1168-4 (PMC5320668; doi:10.1186/s13059-017-1168-4)
Supplement: Additional file 1 — Additional information and figures. Additional information about the statistical method, in particular, the optimization and implementation of epiG, as well as additional figures referenced in the main text. (PDF 1880 kb) [file 13059_2017_1168_MOESM1_ESM.pdf]

EpiG: statistical inference and profiling of DNA methylation from whole-genome bisulphite sequencing data

Martin Vincent*, Kamilla Mundbjerg, Jakob Skou Pedersen, Gangning Liang, Peter A. Jones, Torben Falck Ørntoft, Karina Dalsgaard Sørensen, Carsten Wiuf

February 18, 2017

1 Statistical algorithms and optimisation

A self-mapping T on a space X is a function T from X to X, $T\colon X \to X$. The theoretical basis for optimisation of the posterior likelihood in equation (3) in the main text is the following observation:

Proposition *Let X be a finite discrete space and $f\colon X \to \mathbb{R}$ any function. Consider a collection of self-mappings $T_0, \dots, T_{m-1}$ on X, such that for all $i = 0, \dots, m-1$, and all $x \in X$, it holds that*

(1) $f(T_i x) \geq f(x)$,

(2) If $f(T_i x) = f(x)$ then $T_i x = x$.

Then for any $x_0 \in X$, the iterated sequence $(x_n)_{n \geq 0}$, defined by

$$x_{n+1} = T_{(n \bmod m)} x_n$$

converges in a finite number of steps. Moreover, the limit of $(x_n)_{n\geq 0}$ is a simultaneous fixed point of the self-mappings $T_0, \dots, T_{m-1}$.

Note that every point in the set $\arg\max_{x \in X} f(x)$, that is, the set of $x \in X$ for which $f(x)$ attains its maximum, is a simultaneous fixed point of the self-mappings $T_0, \dots, T_{m-1}$.

1.1 Algorithm for maximisation of the haplotype structure posterior likelihood

In this section we describe the proposed algorithm for finding an approximate maximiser $(\widehat{B}, \widehat{G}, \widehat{R})$ of the posterior likelihood

$$\Lambda(B, G, R) = \pi_0(B)\pi_1(G \,|\, B)\mathcal{L}(B, G, R), \tag{1}$$

which is equation (3) in the main text. The algorithm is guaranteed to converge in a finite number of steps due to the proposition above, but may (in rare cases) only

*Address for correspondence: martin.vincent.dk@gmail.com

return a near optimal solution. We observe that a maximiser of (1) can be found by first finding a maximiser $\widehat{B}$ of

$$\log \widetilde{\Lambda}(B) = \log \pi_0(B) + \max_{G,R}(\log \pi_1(G \,|B) + \log \mathcal{L}(B, G, R)) \tag{2}$$

and then a maximiser $(\widehat{G}, \widehat{R})$ of

$$\log \widetilde{\Lambda}(G, R \mid \widehat{B}) = \log \pi_1(G \,|\widehat{B}) + \log \mathcal{L}(\widehat{B}, G, R). \tag{3}$$

For a given haplotype structure $\widehat{B}$ the maximizer of the term (3) can be computed directly/easily. This means that the optimal epigenotype assignment $\widehat{G}$ and strand assignment $\widehat{R}$ may subsequently be found from (3). Hence, the primary problem is to find a maximiser of the objective (2). The reason for splitting the task into two optimisation problems is that we do not need store the current estimates of (G, R) as they are easily found from (3).

In (2) we are optimising over the space X of haplotype structures. Each haplotype chain is indexed by a number. With n being the total number of reads, the maximal number of haplotype chains is n (one chain for each read), we may, therefore, take $X = \{1, \ldots, n\}^n$.

Let $\widehat{\mathcal{B}_i} \subseteq \{1, \ldots, n\}$ denote the set of feasible haplotype chains overlapping read i, and note that this include the current haplotype chain of read i. For a haplotype structure $B = (b_1, \ldots, b_n) \in X$, define

$$\mathcal{B}_i(B) = \widehat{\mathcal{B}_i} \cup \left(\{1, \ldots, n\} \setminus \bigcup_{j=1}^{n} \{b_j\} \right),$$

where $M_1 - M_2$ is the set difference between two sets M_1 and M_2. The set $\mathcal{B}_i(B)$ is the set of indices of haplotype chains overlapping read i, in addition to those indices that are currently not used.

For $\tilde{b} \in \mathcal{B}_i(B)$ the haplotype structure $(b_1, \ldots, b_{i-1}, \tilde{b}, b_{i+1}, \ldots, b_n)$ may not be feasible, even if B is feasible, since the removal of read i from chain b_i may destroy the feasibility of the chain, for example by creating a "hole" in the chain, that is, a position $j \in [s_b, s_b + L_b]$ with read depth $c(b_i, j) = 0$. The feasibility may be corrected by splitting the haplotype chain b_i into two or more feasible chains. We denote the corresponding self-mapping by S_i and the set of feasible candidate haplotype structures by

$$F_i(B) = \{S_i(b_1, \ldots, b_{i-1}, b, b_{i+1}, \ldots, b_n) \mid b \in \mathcal{B}_i(B)\}.$$

An optimisation procedure may be constructed by choosing n self-mappings $B_1^*, \ldots, B_n^*$ on X such that

$$B_i^*(B) \in \operatorname*{arg\,max}_{x \in F_i(B)} \log \widetilde{\Lambda}(x)$$

for $i = 1, \ldots, n$. Note that since $B \in F_i(B)$ we may have $B_i^*(B) = B$. We also note that the ith read is allowed to be reallocated to an existing haplotype chain or to a

new haplotype chain, namely a chain assigned to an element of $\{1,\ldots,n\}-\bigcup_{i=1}^{n}\{b_i\}$. Moreover, the set $F_i(B)$ is a finite and generally relatively small set, hence the computation of these mappings are relatively inexpensive. Then for $i = 1,\ldots,n$, define

$$T_i B = \begin{cases} B & \text{if } B \in \underset{x\in F_i(B)}{\arg\max}\ \log \widetilde{\Lambda}(x) \\ B_i^*(B) & \text{otherwise.} \end{cases}$$

Evidently $T_1,\ldots,T_n$ fulfil condition (1) and (2) in the above proposition. Furthermore, $T_1,\ldots,T_n$ maps feasible haplotype structures onto feasible haplotype structures.

If we generate a sequence by applying these self-mappings, then by the above proposition, it converges to a simultaneous fixed point of $T_1,\ldots,T_n$ in a finite number of steps. Hence, our algorithm will return a feasible haplotype structure B which is optimal in the sense that moving a single read to another chain, and ensuring feasibility of the haplotype structure implied by this move, then the posterior likelihood Λ will decrease.

2 Implementation and configuration

An implementation of epiG is available as an R package `epiG`. Currently, there is a version on GitHub

`https://github.com/vincent-dk/epiG`.

It can be installed using the R command:

```
# install.packages("devtools")
devtools::install_github("vincent-dk/epiG-pkg")
```

Essential scripts used for data analysis and information on how to use `epiG` is available on GitHub

`github.com/vincent-dk/using-epiG`.

2.1 A simple example

For standard use, `auto_config` will generate the standard configuration for WGBS and for NOMe-seq data, see a simple example in Listing 1. The method `chain_info` retrieve information about the inferred haplotype chains.

2.2 Configuration and conversion models

The statistical conversion model, that is the distribution $\mathbf{P}(Z = z \mid g, r)$, is fully configurable. In normal running mode the same statistical conversion model is applied to all position and the position dependent base-calling reliability being incorporated through equation (1) in the main text. However, the model can be made context dependent (this feature is not used in the examples in the paper, except for NOMe-seq data). The distribution is specified by lists of matrices.

The standard bisulphite conversion model can be generated using the command:

```
model <- BSeq()
```

Listing 1 Simple example of using the epiG package

```
# Load epiG package
library(epiG)

# Specify site (GNAS)
chr <- "chr20"
start <- 57380000
end <- 57478000

# Create epiG configuration
config <- auto_config(
  bam_file = "SRX332736_sorted.bam",
  ref_file = "hg19_rCRSchrm.fa",
  alt_file = "dbsnp_135.hg19.fa",
  chr = chr,
  start = start,
  end = end)

# Run epiG
g <- epiG(max_threads = 3, config = config)

# Load additional data into model
g <- fetch_reads(g) # Load reads
g <- fetch_ref(g) # Load reference genome
g <- fetch_alt(g) # Load alternative nucleotides

# Print a summary of the inferred epigenomic haplotypes
g
```

Listing 2 Information about haplotype chains

```
> cinfo <- chai_info(g)
> cinfo

  chain.id    start      end length nreads nreads.fwd nreads.rev depth.fraction
1        1 57415050 57415300    251     10          3          7    0.036822492
2        2 57415050 57415300    251    182         69        113    0.671733756
3        3 57415050 57415070     21      2          0          2    0.125156446
4        4 57415050 57415300    251    127         56         71    0.468398332
5        5 57415115 57415289    175      2          2          0    0.009851731
6        6 57415131 57415296    166      2          2          0    0.010417752
7        7 57415132 57415284    153      2          0          2    0.011361054
```

Listing 3 Information about inferred genotype

```
> info <- position_info(g, 57415143 + c(0,1))
> info[, c("position", "chain.id", "ref", "genotype", "fit.ratio",
 "methylated", "nreads.fwd", "nreads.rev")]

   position chain.id ref genotype     fit.ratio methylated nreads.fwd nreads.rev
1  57415143        1   C        C   -37.5505611         NA          0          2
2  57415143        2   C        C  -898.5909394       TRUE         21         35
4  57415143        4   C        C  -336.3580702      FALSE         20         18
5  57415143        5   C        C   -20.9756797       TRUE          1          0
6  57415143        6   C        C   -20.0545543       TRUE          1          0
7  57415143        7   C        C   -20.1571365         NA          0          1
11 57415144        1   G        G    -0.7762233      FALSE          0          2
21 57415144        2   G        G  -909.3393081       TRUE         21         34
41 57415144        4   G        G  -334.5881731      FALSE         19         18
51 57415144        5   G        G   -21.0782635         NA          1          0
61 57415144        6   G        G   -21.0782635         NA          1          0
71 57415144        7   G        G     0.1025810      FALSE          0          1
```

The BSeq function allows the user to specify the failed bisulphite conversion rate (α) the inappropriately conversion rate (β) and the maximal read length, with

standard values listed in Table S1. The function will generate two lists of matrices; one list for forward reads and one for reverse reads, see Listing 5.

Listing 4 Information about reads

```
> rinfo <- read_info(g)
> rinfo

                    name    start      end length read.id chain.id strand
1   SRR1609024.193786394 57414951 57415051    101       1        2    rev
2   SRR1609023.192925911 57414954 57415052     99       2        2    fwd
3   SRR1609025.194393042 57414954 57415054    101       3        2    rev
4   SRR1609025.194393051 57414954 57415054    101       4        2    rev
5   SRR1609025.194393037 57414956 57415054     99       5        2    rev
6   SRR1609023.192925914 57414958 57415058    101       6        2    rev
7   SRR1609024.193786395 57414960 57415060    101       7        2    rev
8   SRR1609024.193786397 57414960 57415060    101       8        2    rev
9   SRR1609024.193786389 57414967 57415065     99       9        4    fwd
10  SRR1609023.192925912 57414969 57415067     99      10        3    rev

...

322 SRR1609026.195055112 57415295 57415393     99     322        4    fwd
323 SRR1609025.194393075 57415296 57415394     99     323        2    fwd
324 SRR1609025.194393111 57415296 57415396    101     324        2    rev
325 SRR1609025.194393076 57415299 57415397     99     325        2    rev
326 SRR1609023.192925955 57415300 57415398     99     326        4    rev
327 SRR1609024.193786431 57415300 57415398     99     327        4    rev
```

Listing 5 Standard bisulphite conversion matrices

```
model$fwd[[1]]
     C G A T C^me G_me
C 0.05 0 0 0 0.95    0
G 0.00 1 0 0 0.00    1
A 0.00 0 1 0 0.00    0
T 0.95 0 0 1 0.05    0

model$rev[[1]]
  C    G A T C^me G_me
C 1 0.00 0 0    1 0.00
G 0 0.05 0 0    0 0.95
A 0 0.95 1 0    0 0.05
T 0 0.00 0 1    0 0.00
```

In context dependent model different statistical conversion models are applied to different genomic contexts. There are 5 different contexts

1 DGCH (isolated GpC)
2 HCGD (isolated CpG)
3 CH and not DGCH
4 NCN or NGN and not 1-3
5 Other

For example the sequence GCGCACGAGCACAT will be interpreted as the being in the contexts 44435225115355. In context dependent mode 5×2 list of matrices needs to be specified. Context dependent mode is used for NOMe-seq data, where the conversion models for context 1, 2 and 5 are identical to the standard bisulphite conversion model listed above. However, the conversion models for context 3 and 4 are identical, but modified such that CpG methylation is ignored. The conversion matrices for context 4 is given in Listing 6.

The program epiG may further be configured using the `epiG_config` command; see the manual for details.

Listing 6 NOMe-Seq conversion matrices for NCN and NGN context

```
model$fwd_C_G[[1]]
    C G A T C^me G_me
C 0.5 0 0 0  0.5    0
G 0.0 1 0 0  0.0    1
A 0.0 0 1 0  0.0    0
T 0.5 0 0 1  0.5    0

model$rev_C_G[[1]]
  C   G A T C^me G_me
C 1 0.0 0 0    1  0.0
G 0 0.5 0 0    0  0.5
A 0 0.5 1 0    0  0.5
T 0 0.0 0 1    0  0.0
```

3 Running Bis-SNP

We used the the following command and options when running Bis-SNP.

Listing 7 Running Bis-SNP

```
java -Xmx4g -jar BisSNP-0.82.2.jar
  -R hg19_rCRSchrm.fa
  -T BisulfiteGenotyper
  -I LNCaP.recal.bam
  -D dbsnp_135.hg19.sort.vcf
  -stand_emit_conf -10
  -nt 8
  -out_modes EMIT_ALL_SITES
  -vfn1 cpg.raw.vcf
```

Author details

References

1. Fang F, Hodges E, Molaro A, Dean M, Hannon GJ, Smith AD: Genomic landscape of human allele-specific DNA methylation. Proc Natl Acad Sci USA. 2012;109(19):7332–7337.
2. Kelly TK, Liu Y, Lay FD, Liang G, Berman BP, Jones PA: Genome-wide mapping of nucleosome positioning and DNA methylation within individual DNA molecules. Genome Res. 2012;22:2497–2506.
3. Genereux DP, Johnson WC, Burden AF, Stöger R, Laird CD: Errors in the bisulfite conversion of DNA: modulating inappropriate-and failed-conversion frequencies. Nuc Acids Res. 2008;36:150–150.
4. Leontiou CA, Hadjidaniel MD, Mina P, Antoniou P, Ioannides M, Patsalis PC: Bisulfite Conversion of DNA: Performance comparison of different kits and methylation quantitation of epigenetic biomarkers that have the potential to be used in non-invasive prenatal testing. PLoS ONE. 2015;10(8): e0135058.

4 Additional tables and figures

Table S1. Parameters and their default values for WGBS and NOMe-seq data.

Table S2. List of 18 AMRs used in Fig. 5 in the main text, from [1].
The data is filtered for noise as described in the main text.

S1-S18. Analysis of 18 AMRs in Table S2 (18 figures).
Generally, the number and extent/length of haplotype chains in the four samples are highly comparable for all 18 AMRs. The data is filtered for noise as described in the main text. Vertical bars indicate the known regions of ASM in each of the 18 gene regions [1]. These regions are the combined ASM regions compiled from 22 methylomes from cell lines and tissue samples (not including colon) [1].

S19. Periodic pattern in nucleosome occupancy data.
The plots show strong periodicity in nucleosome occupancy with a phasing corresponding roughly to the length of the nucleosome, ∼150bp. CpG methylation shows similar periodicity, except for a 400-500bp CpG methylation repressed region around the CTCF site. Importantly, this validation only tests the position-wise consensus prediction over many sites and does not use the information contained in the inferred haplotype chains.

S20. Noise: depth fraction and length of haplotype chains.
Decision line to filter out noise based on depth fraction and length of the haplotype chain.

S21. Noise in inferred haplotype chains in GNAS locus.

S22. Noise in inferred haplotype chains in H19 gene.

S23. ROC curves for methylation validation, with default parameters $\alpha = 0.95$ and $\beta = 0.05$.
All parameters are put to their default values, see Table 3 in the main text.

S24. Genotype validation, correctly called SNPs.
The figure extends that of Fig. 10 in the main text. SNP genotyping is best for the homozygous genotypes and for AC, CG, TA, TG, and less good for CT, AG. The latter genotypes might be confused with methylation marks.

S25. AUC curves for methylation validation, with varying bisulphite conversion rate (α) and inappropriate conversion rate (β).
The performance of epiG, when measured by AUC, does not changed significantly for other parameter values than the default parameters; the AUC varies between 0.96 to 0.98.

S26. SNP validation and the prior $\pi_1(G\,|B)$.
A high q value in the prior is recommended.

S27. K_0, K_1 parameters.
If the overlap in CpG sites is set to zero ($K_0 = 0$), two reads that overlap but that do not share a CpG site, will be grouped together. Hence reads from biologically different epi-alleles might accidentally be joined.

S28. Read depth for the data used in the main text.
The read depth varies throughout the genome and for the different samples. Read depth is generally very low for LNCaP and PrEC, while significantly higher for the other samples (see also Table 4 in the main text).

Name	Description	WGBS (single)	WGBS (paired)	NOMe-seq (paired/single)
bisulfite_rate	Bisulfite conversion rate	0.95	0.95	0.95
bisulfite_inap.rate	Bisulfite inappropriate conversion rate	0.05	0.05	0.05
min_overlap_length	Minimum overlapping length	40	50	40
min_CG_count	Minimum overlapping CG positions	1	2	0
min_HCGD_count	Minimum overlapping HCGD positions	0	0	0
min_DGCH_count	Minimum overlapping DGCH positions	0	0	2
ref_prior	Genotype prior parameter	0.9999	0.9999	0.9999
margin	Cut off margin	5	5	5
use_paired.reads	Use paired reads	FALSE	TRUE	TRUE/FALSE
context_dependent	Context dependent mode	FALSE	FALSE	TRUE
max_iterations	Max number of iterations	1e5	1e5	1e5
quality_threshold	Read mean epsilon quality threshold	0.020	0.020	0.020

Table S1 Parameters and their default values for WGBS and NOMe-seq data.

Chromosome	Region (hg19)		Gene/locus	Reference
1	68515537	68517691	LOC100289178	Yu et al. (1999)
4	89617864	89619549	HERC3	Monk et al. (2011)
7	130129559	130133682	MESTIT1, MIR335, MEST	Kobayashi et al. (1997)
7	94284018	94289851	SGCE, PEG10	One et al. (2001)
7	27179065	27187138	HOXA6, HOXA5, LOC100133311	Strathdee et al. (2006)
10	127584249	127588031	FANK1	Li et al. (2010)
11	10527505	10531695	AMPD3, RNF141	Schultz et al. (2006)
11	2719386	2722440	KCNQ1	Horike et al. (2000)
11	2016476	2024739	H19, MIR675	Licifer et al. (2004)
14	101290239	101295152	MEG3	Rocha et al. (2008)
15	25199298	25202152	SNRPN, SNURF	Sutcliffe et al. (1994)
16	3492753	3494769	ZNF597, NAA60, NAT15	Nakabayashi et al. (2011)
19	57348719	57353128	MIMT1, ZIM2, PEG3	Huang et al. (2009)
20	57414161	57418015	GNAS-AS1	Frohlich et al (2010)
20	57424981	57431470	GNAS-AS1, GNAS	Frohlich et al (2010)
20	30134590	30135902	HM13	Monk et al. (2011)
20	57463105	57465570	GNAS	Frohlich et al (2010)
20	36148274	36151269	BLCAP, NNAT	Evans et al. (2001)

Table S2 Known allele-specific methylated regions (AMRs) used in this study; see Additional figure S1-S18 and [1] for further details. Gene/locus assignment is taken from [1] and not the original publications.

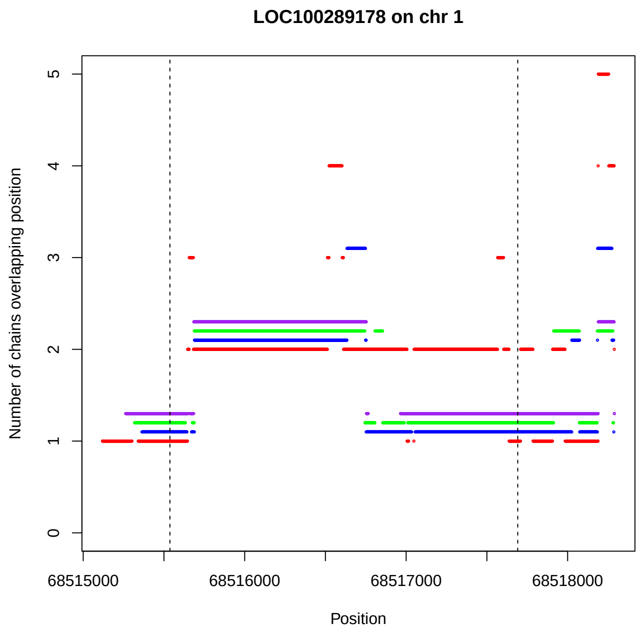

Figure S1 LOC100289178. The figure shows the number of haplotype chains overlapping a particular position. Dashed lines indicate the AMR, see [1] for further details. Red: Colon normal A, Purple: Colon normal B, Blue: Colon tumor B, Green: WA9.

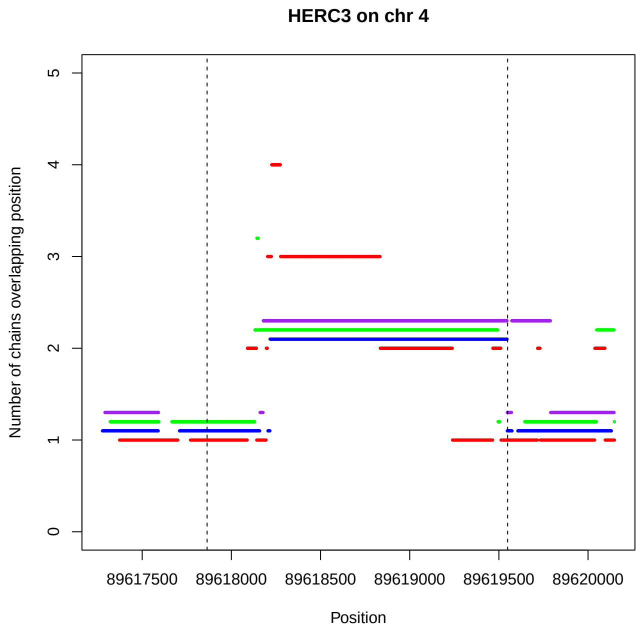

Figure S2 HERC3. The figure shows the number of haplotype chains overlapping a particular position. Dashed lines indicate the AMR, see [1] for further details. Red: Colon normal A, Purple: Colon normal B, Blue: Colon tumor B, Green: WA9.

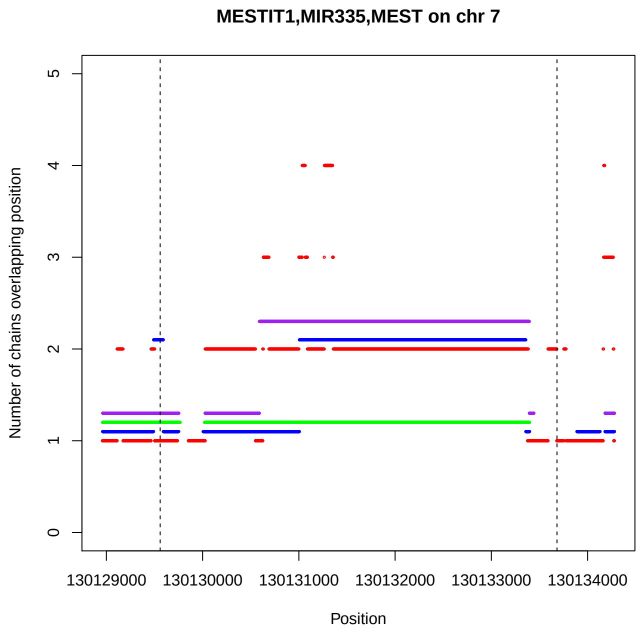

Figure S3 **MESTIT1, MIR335, MEST.** The figure shows the number of haplotype chains overlapping a particular position. Dashed lines indicate the AMR, see [1] for further details. Red: Colon normal A, Purple: Colon normal B, Blue: Colon tumor B, Green: WA9.

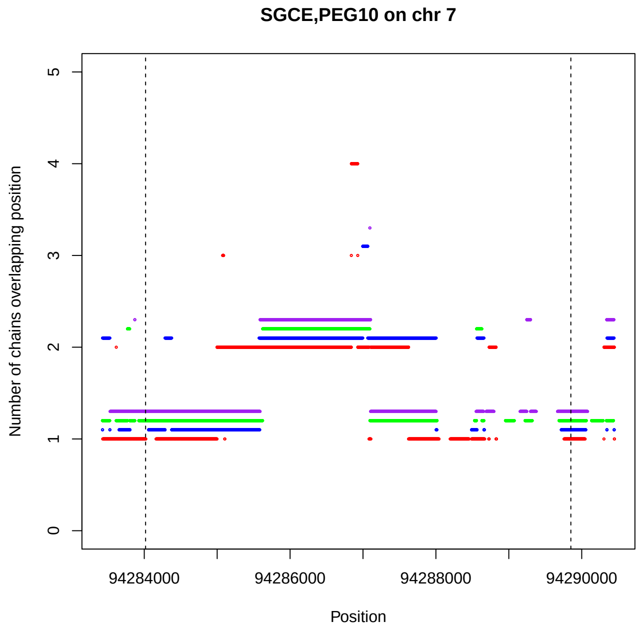

Figure S4 **SGCE, PEG10.** The figure shows the number of haplotype chains overlapping a particular position. Dashed lines indicate the AMR, see [1] for further details. Red: Colon normal A, Purple: Colon normal B, Blue: Colon tumor B, Green: WA9.

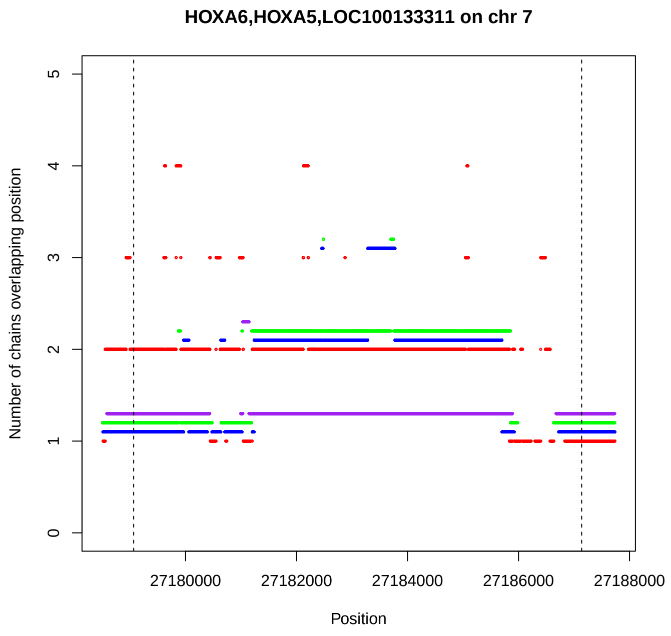

Figure S5 HOXA6, HOXA5, LOC100133311. The figure shows the number of haplotype chains overlapping a particular position. Dashed lines indicate the AMR, see [1] for further details. Red: Colon normal A, Purple: Colon normal B, Blue: Colon tumor B, Green: WA9.

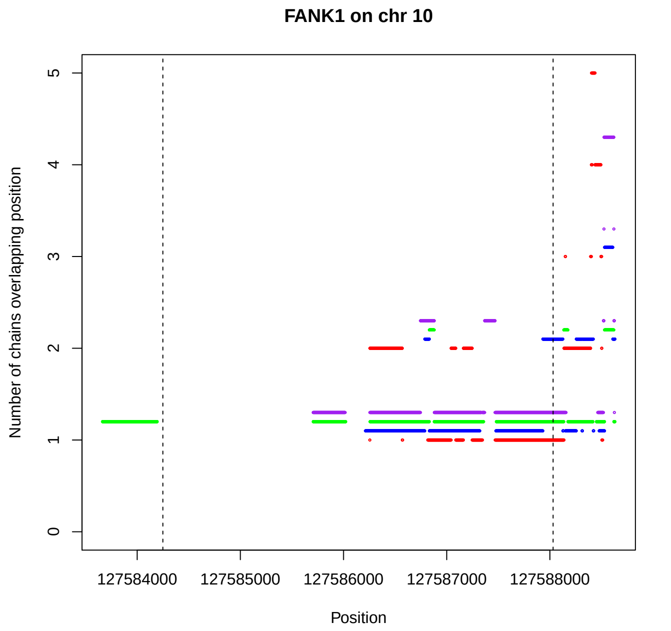

Figure S6 FANK1. The figure shows the number of haplotype chains overlapping a particular position. Dashed lines indicate the AMR, see [1] for further details. Red: Colon normal A, Purple: Colon normal B, Blue: Colon tumor B, Green: WA9.

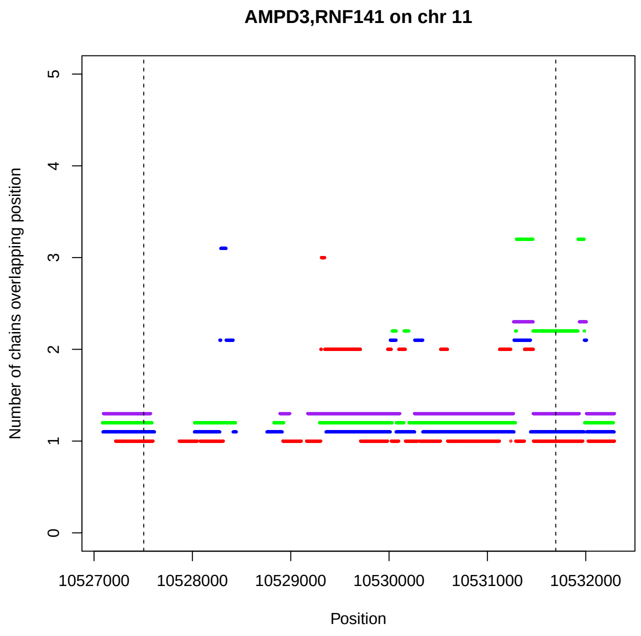

Figure S7 **AMPD3, RNF141.** The figure shows the number of haplotype chains overlapping a particular position. Dashed lines indicate the AMR, see [1] for further details. Red: Colon normal A, Purple: Colon normal B, Blue: Colon tumor B, Green: WA9.

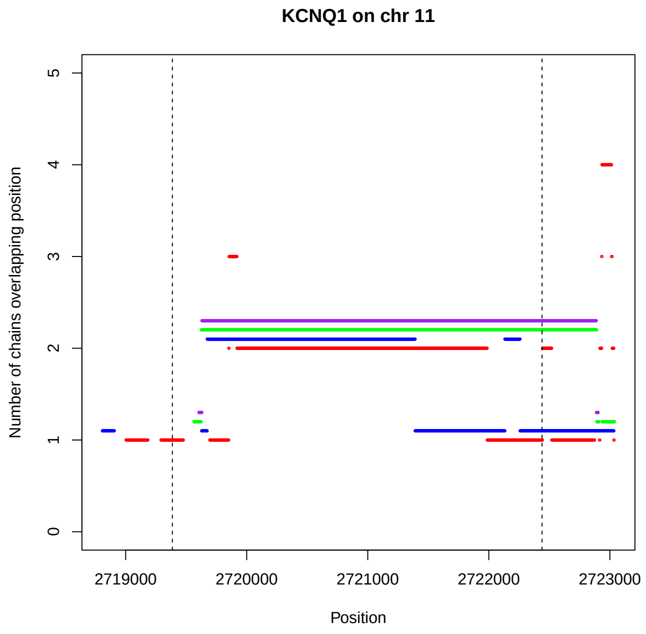

Figure S8 **KCNQ1.** The figure shows the number of haplotype chains overlapping a particular position. Dashed lines indicate the AMR, see [1] for further details. Red: Colon normal A, Purple: Colon normal B, Blue: Colon tumor B, Green: WA9.

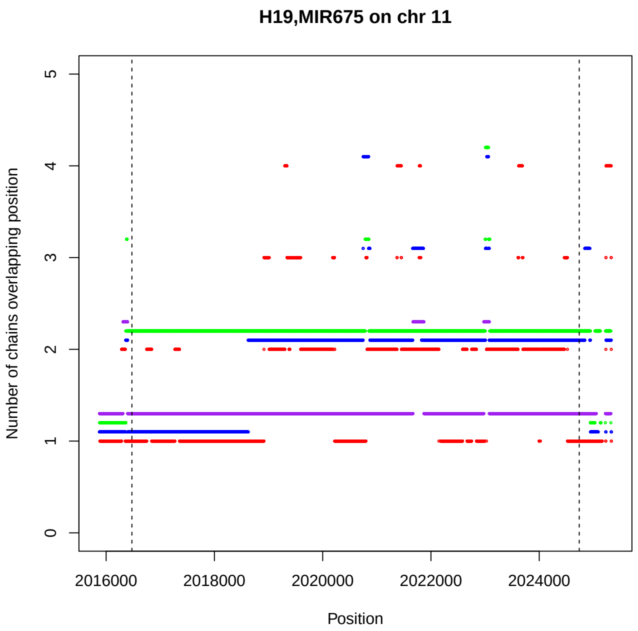

Figure S9 **H19, MIR675.** The figure shows the number of haplotype chains overlapping a particular position. Dashed lines indicate the AMR, see [1] for further details. Red: Colon normal A, Purple: Colon normal B, Blue: Colon tumor B, Green: WA9.

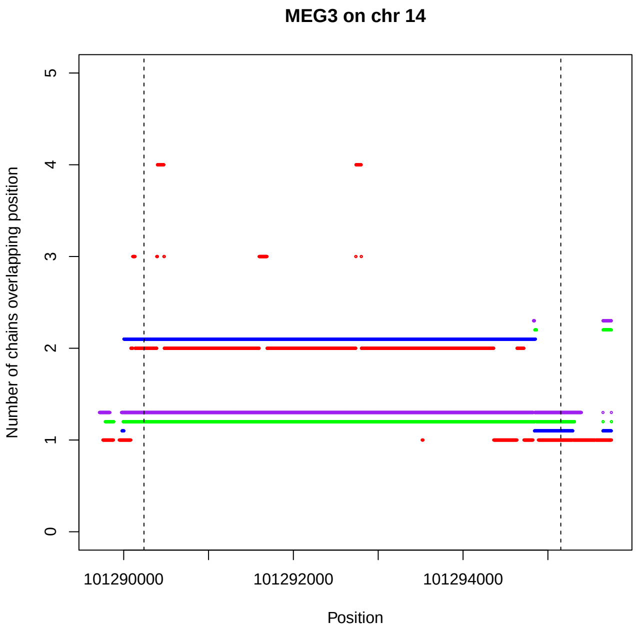

Figure S10 **MEG3.** The figure shows the number of haplotype chains overlapping a particular position. Dashed lines indicate the AMR, see [1] for further details. Red: Colon normal A, Purple: Colon normal B, Blue: Colon tumor B, Green: WA9.

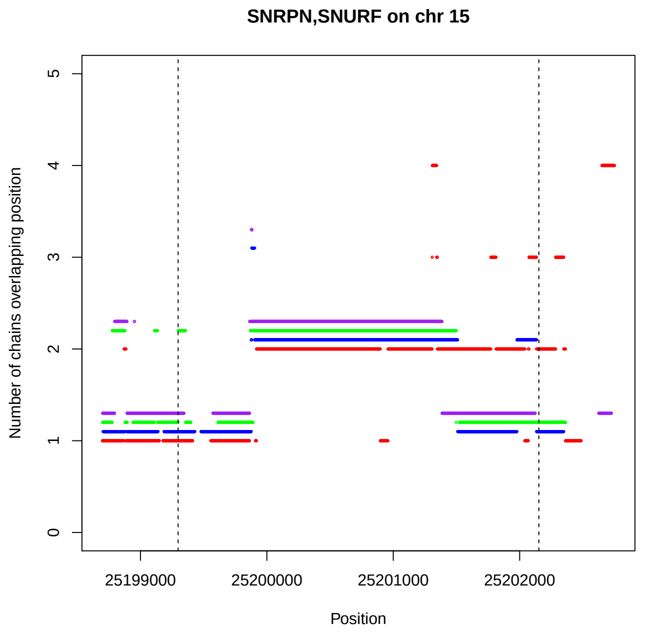

Figure S11 **SNRPN, SNURF.** The figure shows the number of haplotype chains overlapping a particular position. Dashed lines indicate the AMR, see [1] for further details. Red: Colon normal A, Purple: Colon normal B, Blue: Colon tumor B, Green: WA9.

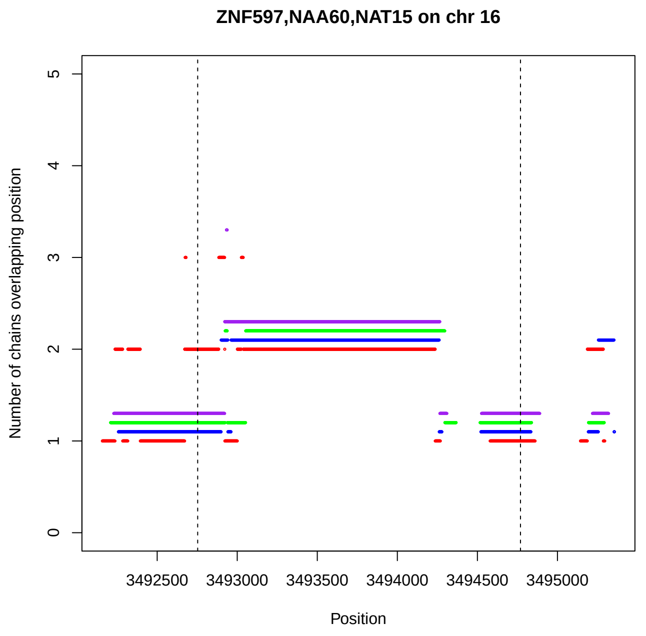

Figure S12 **ZNF597, NAA60, NAT15.** The figure shows the number of haplotype chains overlapping a particular position. Dashed lines indicate the AMR, see [1] for further details. Red: Colon normal A, Purple: Colon normal B, Blue: Colon tumor B, Green: WA9.

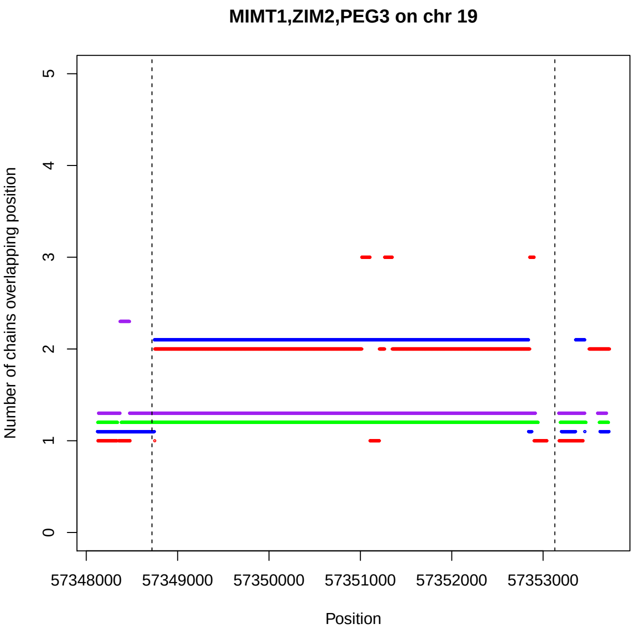

Figure S13 **MIMT1, ZIM2, PEG3.** The figure shows the number of haplotype chains overlapping a particular position. Dashed lines indicate the AMR, see [1] for further details. Red: Colon normal A, Purple: Colon normal B, Blue: Colon tumor B, Green: WA9.

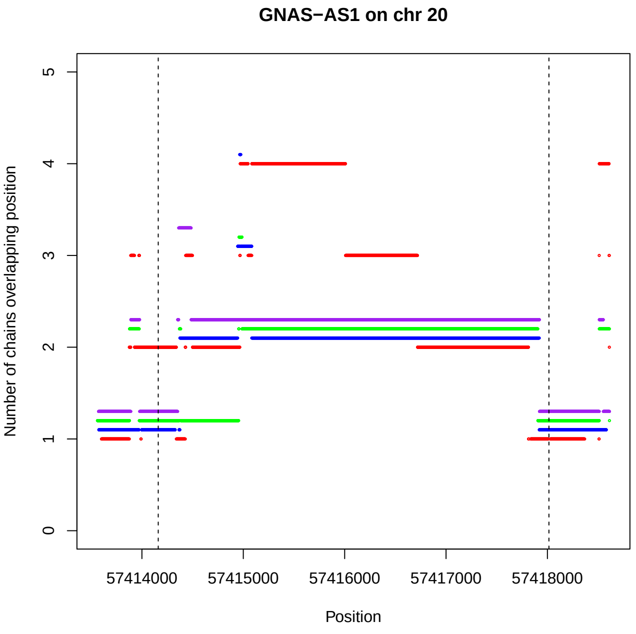

Figure S14 **GNAS-AS1.** The figure shows the number of haplotype chains overlapping a particular position. Dashed lines indicate the AMR, see [1] for further details. Red: Colon normal A, Purple: Colon normal B, Blue: Colon tumor B, Green: WA9.

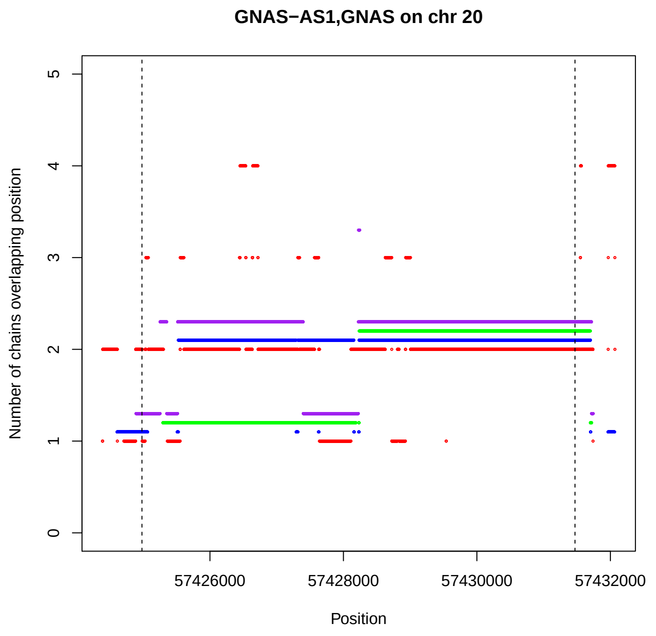

Figure S15 GNAS-AS1, GNAS. The figure shows the number of haplotype chains overlapping a particular position. Dashed lines indicate the AMR, see [1] for further details. Red: Colon normal A, Purple: Colon normal B, Blue: Colon tumor B, Green: WA9.

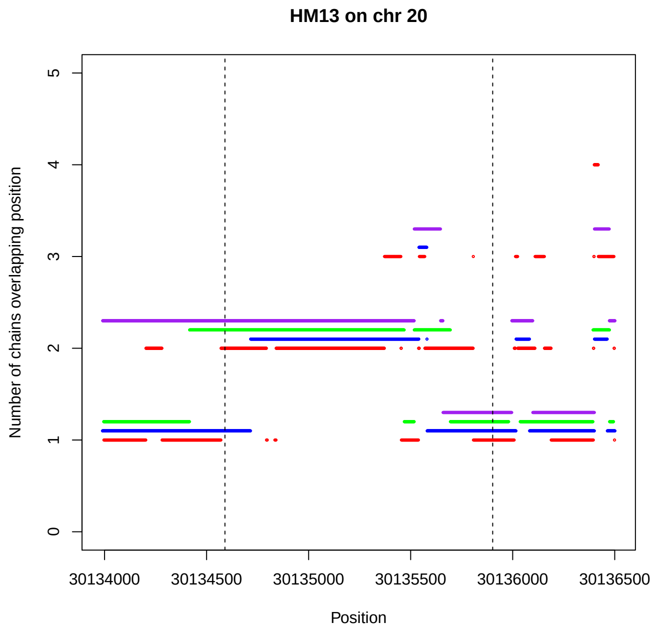

Figure S16 HM13. The figure shows the number of haplotype chains overlapping a particular position. Dashed lines indicate the AMR, see [1] for further details. Red: Colon normal A, Purple: Colon normal B, Blue: Colon tumor B, Green: WA9.

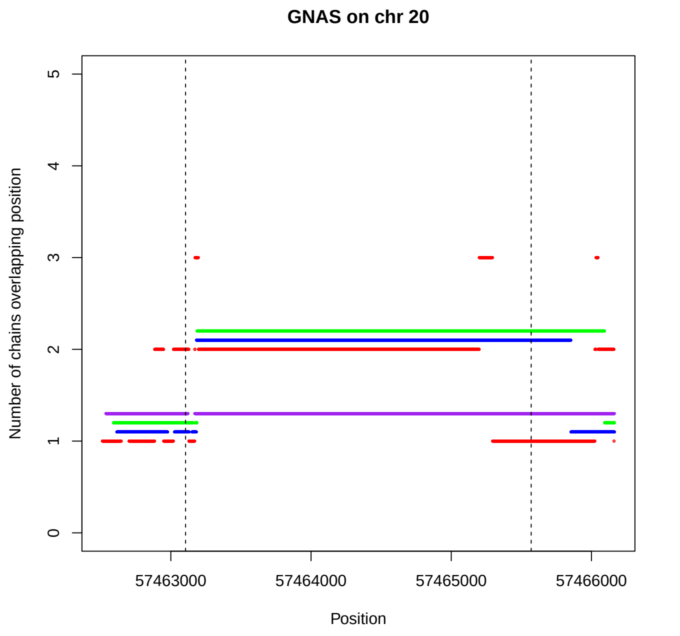

Figure S17 GNAS. The figure shows the number of haplotype chains overlapping a particular position. Dashed lines indicate the AMR, see [1] for further details. Red: Colon normal A, Purple: Colon normal B, Blue: Colon tumor B, Green: WA9.

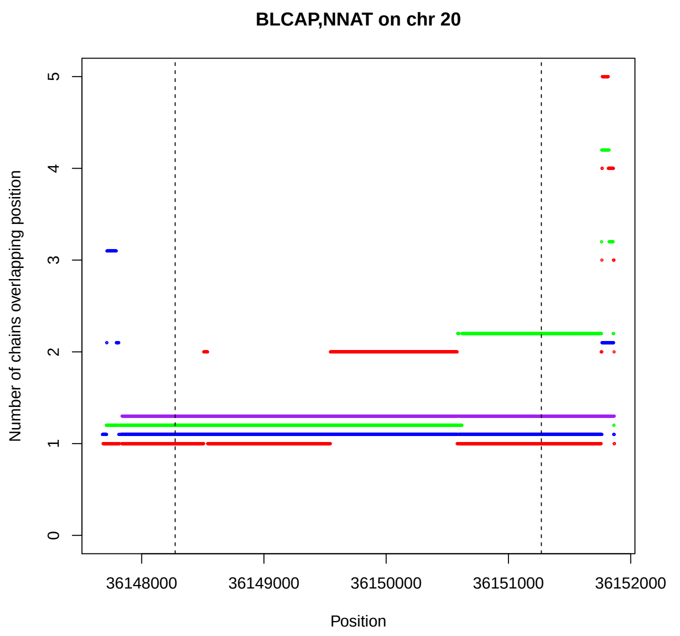

Figure S18 BLCAP, NNAT. The figure shows the number of haplotype chains overlapping a particular position. Dashed lines indicate the AMR, see [1] for further details. Red: Colon normal A, Purple: Colon normal B, Blue: Colon tumor B, Green: WA9.

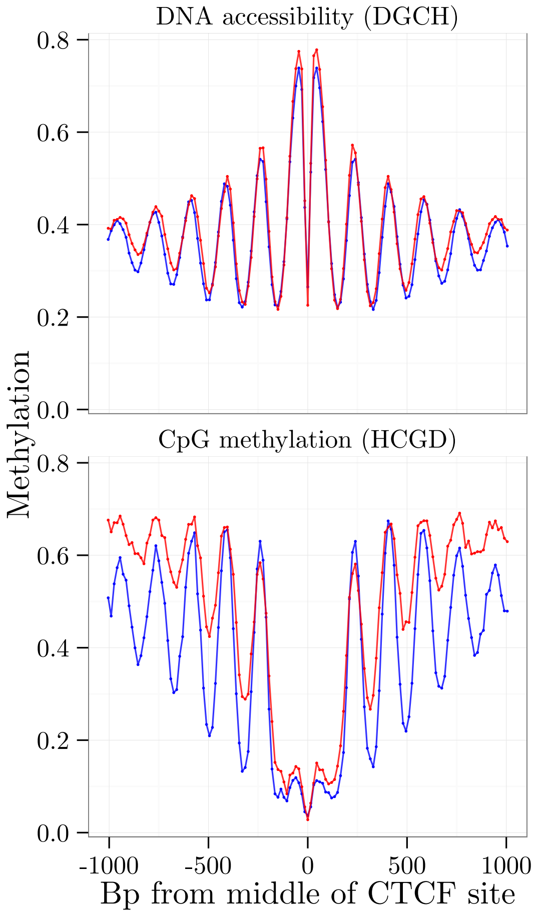

Figure S19 **Average CpG and GpC methylation near CTCF sites.** Top: average methylation level at DGCH positions plotted against the distance from the CTCF site, using the same collection of CTCF sites as in [2]. Bottom: average methylation level at HCGD positions plotted against the distance from the CTCF site. Blue curve: LNCaP. Red curve: PrEC. Methylation levels are generally lower in PrEC than in LNCaP. The average methylation level is computed within windows of length 15bp.

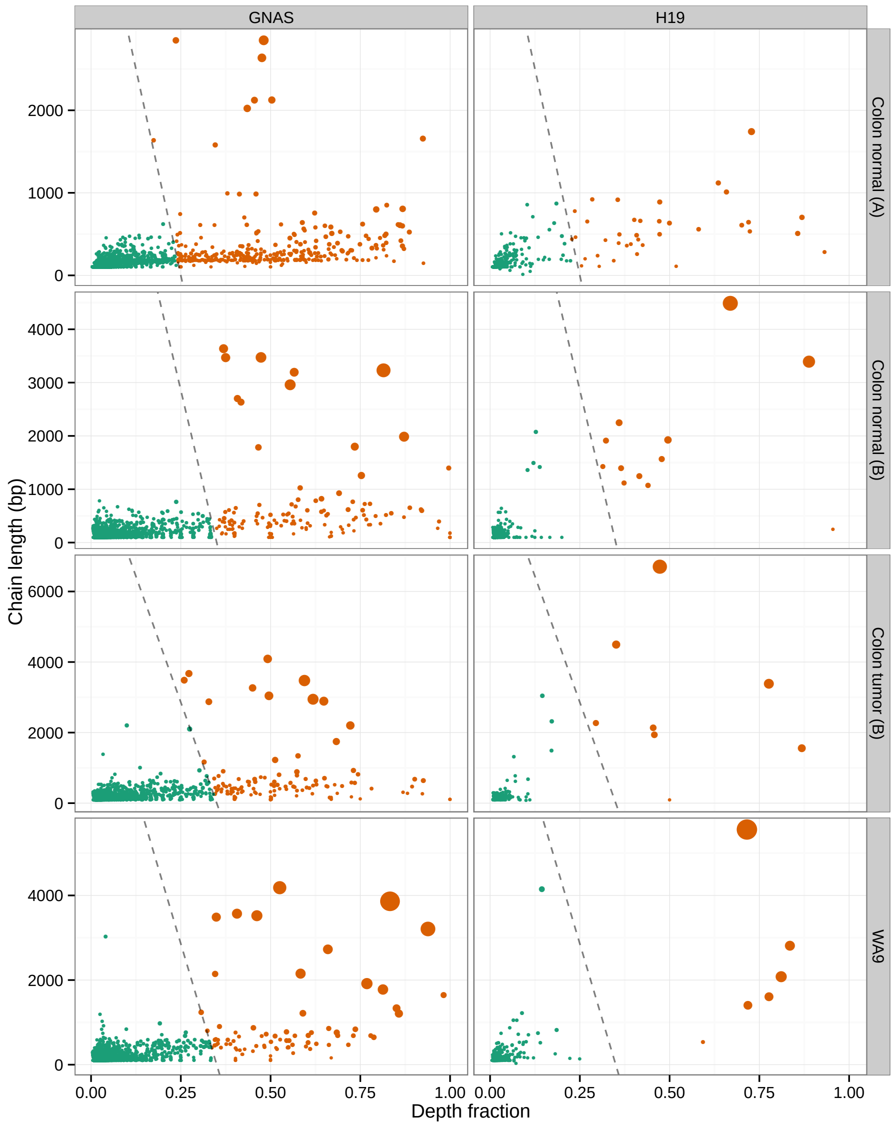

Figure S20 Depth fraction and length of haplotype chains. Plots showing the lengths of haplotype chains against the depth fraction of the haplotype chains for four samples (Colon normal A, Colon normal B, Colon tumor B and WA9) and two gene regions (GNAS locus and gene *H19*). Each point is a chain, the size indicating the number of reads in the chain. In order to reduce noise, haplotype chains falling to the left of the straight line in each plot are removed as they either have short length or contain a low fraction of the reads in the region. Green: removed chains. Brown: kept chains. The exact same cut-off was applied to all samples and analyses (note the scales in the plots differ between them, hence the dashed lines appear different). The haplotype chains are plotted against the genomic position in Additional figure S21 (GNAS locus) and Additional figure S22 (*H19*), and in Fig. 3 (GNAS locus) and Fig. 4 (*H19*) in the main text, after removal of noise. Note that the main determinant for removing chains is the depth fraction. Also note that by changing the criteria for filtering out chains (the straight line), we do not alter the number of dominating chains (unless of course, the line is moved far to the right).

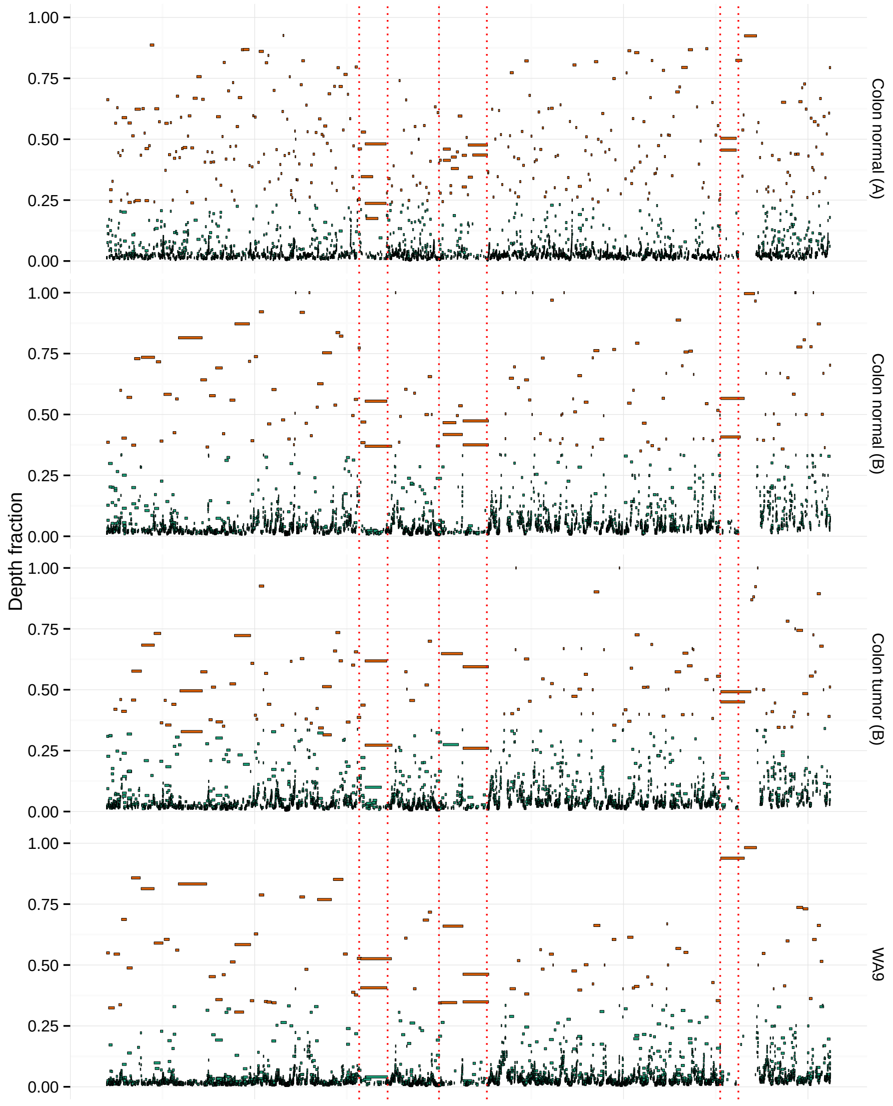

Figure S21 Inferred haplotype chains in the GNAS locus. The plot shows all inferred haplotype chains in a 100kb region near the GNAS locus, with three known ASM regions (vertical red lines), for different samples and before noise reduction (see Fig. 3 in the main text for after noise reduction). The depth fraction of a chain is plotted against its genomic position. The colour indicates whether the chain is kept (brown) or interpreted as noise (green), see Additional figure S20.

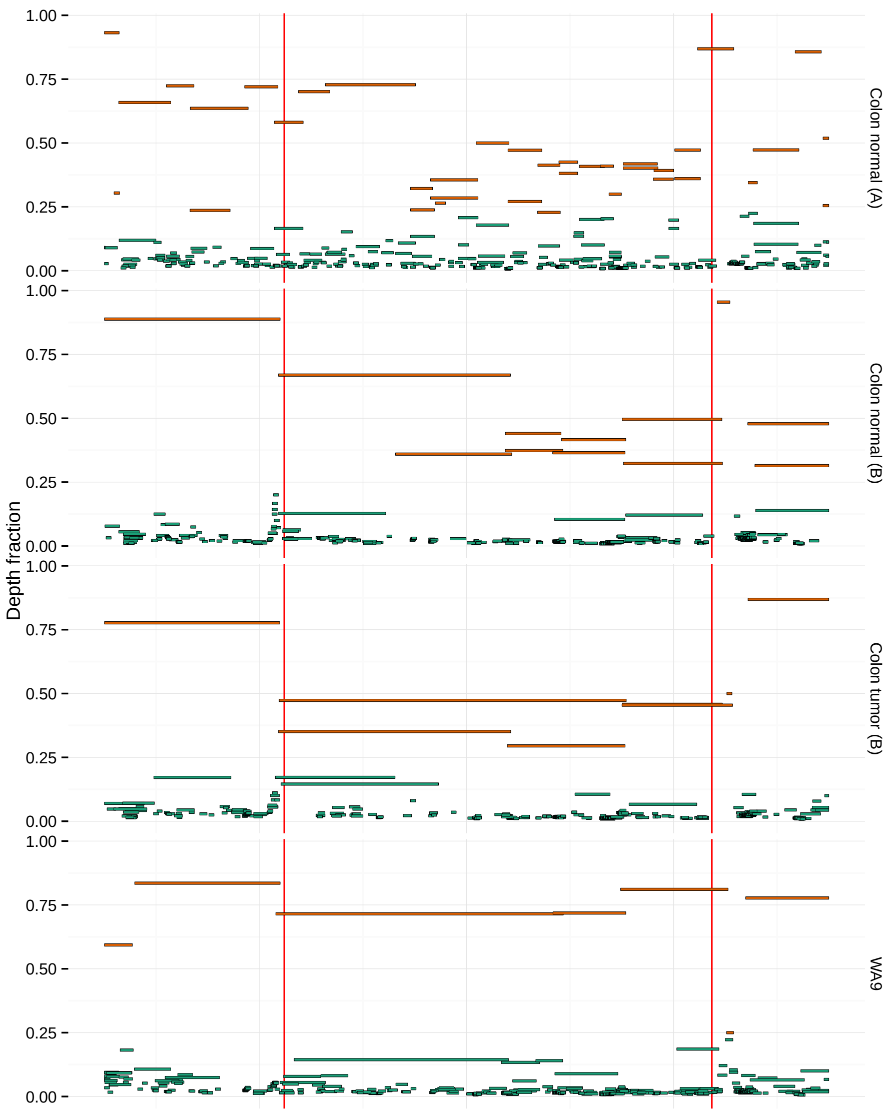

Figure S22 Inferred haplotype chains in H19 gene. The plot shows all inferred haplotype chains in a 20kb region around the H19 gene with one known ASM region (vertical red lines) for different samples and before noise reduction (see Fig. 4 in the main text for after noise reduction). The depth fraction of a chain is plotted against its genomic position. The colour indicates whether the chain is kept (brown) or interpreted as noise (green), see Additional figure S20.

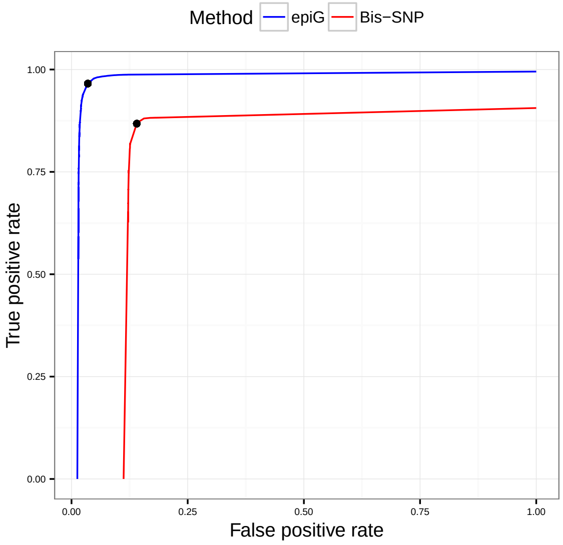

Figure S23 ROC curves for methylation validation, with default parameters $\alpha = 0.95$ and $\beta = 0.05$. The ROC curves are based on 18k isolated CpG nucleotides in the LNCaP genome as described in the main text, section 'Methylation validation'. The optimal predictors are shown in black. Inference might fail for epiG and Bis-SNP if there are insufficient data. It happens for 10.2% of the data for Bis-SNP and 0.8% for epiG, which is the reason the curves do not extend to the points (0,0) and (1,1). Blue: epiG, Red: Bis-SNP.

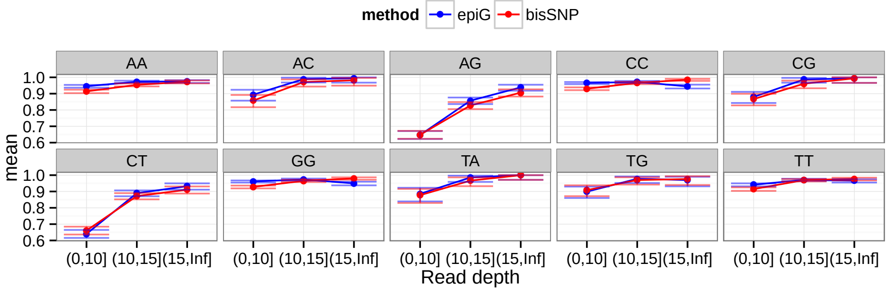

Figure S24 Correctly called SNPs, with default parameters. The figure shows the average number of correctly called SNPs as a function of read depth and true genotype. The figure extends that of Fig. 10 in the main text. The call of the Affymetrix SNP6.0 arrays is taken as the truth. The method for calculating the correctly called SNPs is described in the section 'Genotype validation' in the main text. CT and AG are harder to predict because of potential confusion with CpG methylation. Blue: epiG, Red: Bis-SNP.

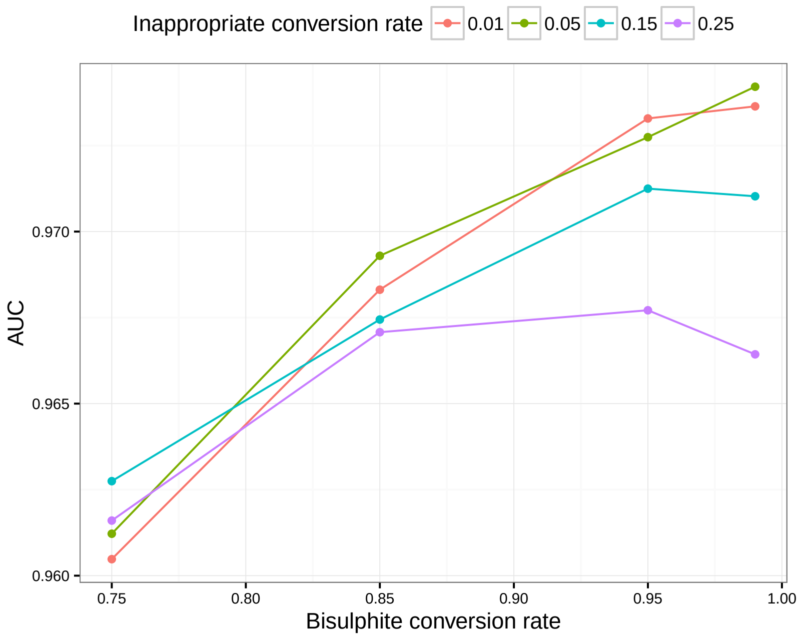

Figure S25 **AUC curves for methylation validation, with varying bisulphite conversion rate (α) and inappropriate conversion rate (β).** The AUC curves are based on 18k isolated CpG nucleotides in the LNCaP genome with varying bisulphite conversion rate ($\alpha = 0.75, 0.85, 0.95, 0.99$) and inappropriate conversion rate ($\beta = 0.01, 0.05, 0, 15, 0.25$). Default parameters are $\alpha = 0.95$ and $\beta = 0.05$. All other parameters are put to their default values. In the investigated range of parameter values, the performance of epiG varies by $\approx 2\%$ in AUC values; hence the performance is largely unaffected by changes in the parameter values, yielding high robustness.

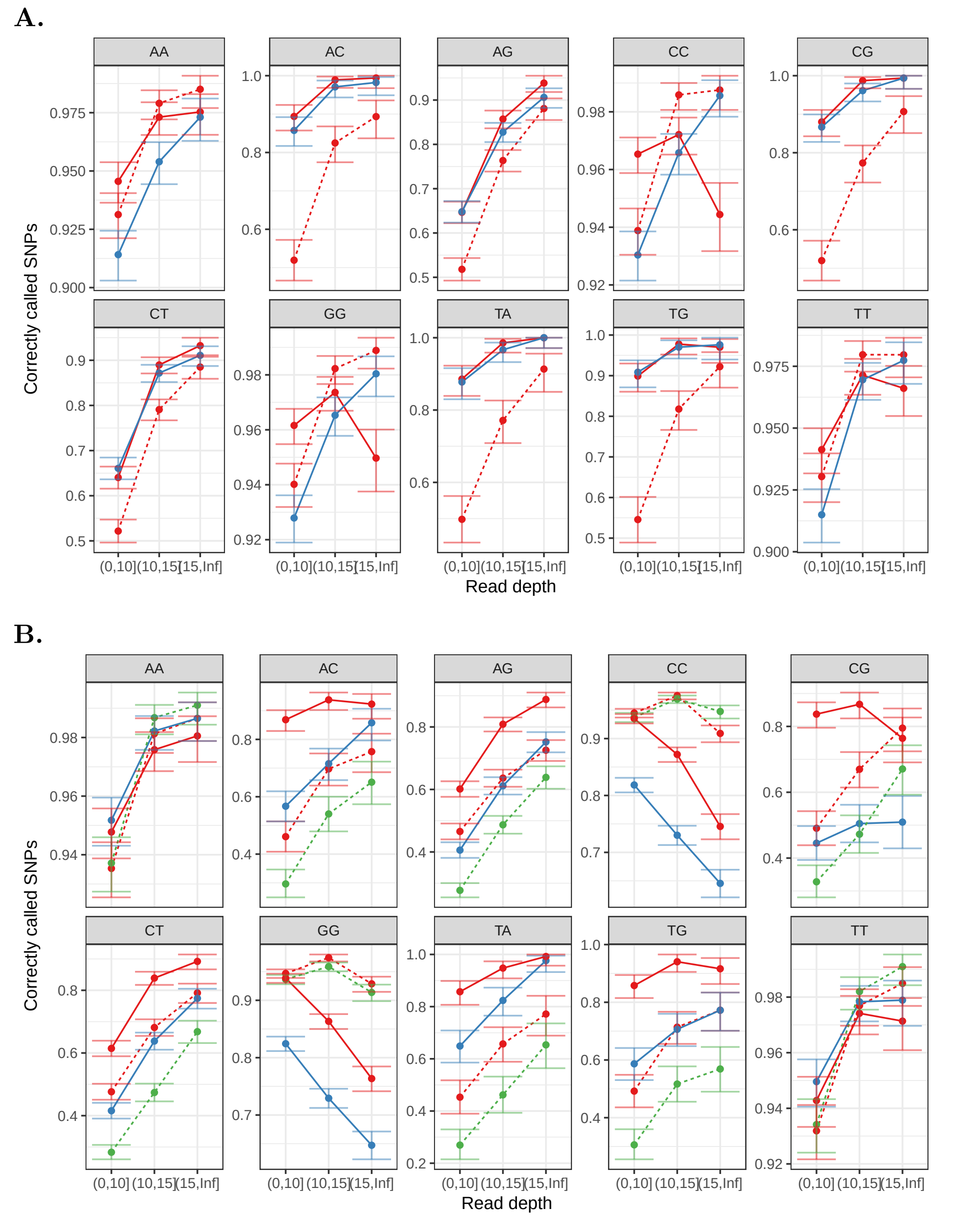

Figure S26 **Genotype validation and the prior $\pi_1(G|B)$.** We use ∼74k SNPs for genotype validation, as described in the main text. The curves correspond to different configurations of the genotype prior (excluding/including dbSNP135 and choosing q). **A.** Red: epiG with $q = 0.9999$ (default setting); Blue: Bis-SNP. **B.** Red: epiG with $q = 0.90$; Blue: epiG with $q = 0.75$; Green: epiG with $q = 0.50$. Dashed lines: without dbSNP135; solid lines: with dbSNP135 (default). The reference genome h19 is always used, also by Bis-SNP. The performance generally declines with decreasing q. For the heterozygous genotypes, the performance is generally lower without dbSNP135 than with dbSNP135. For the genotypes CC and GG, epiG might confuse the true homozygous genotype with a heterozygous genotype because of ambiguities introduced by the bisulphite conversion process. For example, assume we study a CT SNP, and that the individual under consideration has genotype CC. If and one or both of the Cs are methylated, then C and/or T will be observed in the reads (with higher read depth we are more likely to observe both nucleotides). If dbSNP135 is used (solid lines), then C (T) will be in hg19 (dbSNP135), or T (C) will be in hg19 (dbSNP135). This would not be a problem if dbSNP135 is not used.

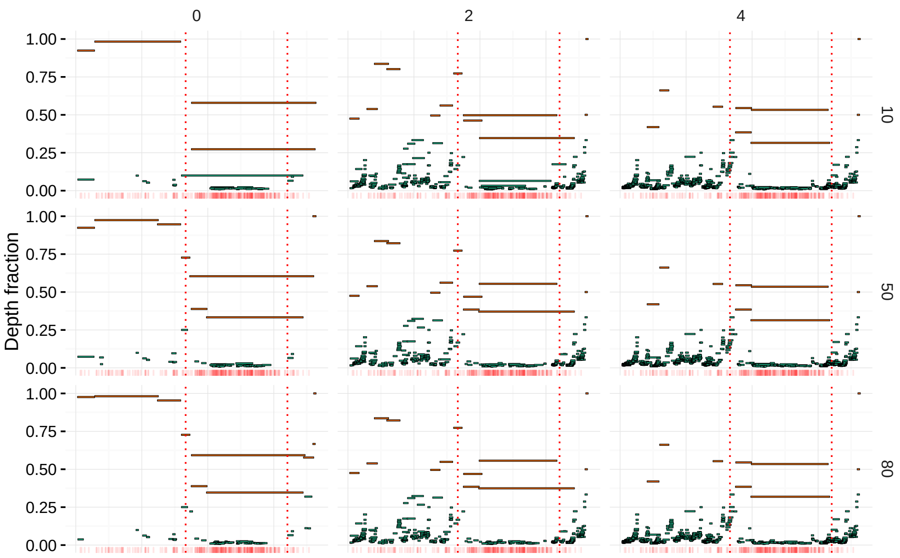

Figure S27 **The parameters K_0, K_1.** The plot shows all inferred haplotype chains of the Colon normal B sample in a 10kb region in the GNAS locus, overlapping one known ASM region (vertical red lines), for different configurations of the parameters K_0 and K_1 and before noise reduction. The depth fraction of a chain is plotted against its genomic position. If $K_0 = 0$, two reads might be joined without sharing a common CpG, thereby creating long epi-alleles that might not exist biologically. $K_0 = 2, 4$ give similar results and these are largely independent of K_1. The colour indicates whether the chain is kept (brown) or interpreted as noise (green). The top numbers are K_1 values (minimum number of CpG overlaps), the number on the right side are K_0 values (minimum number of bp overlaps).

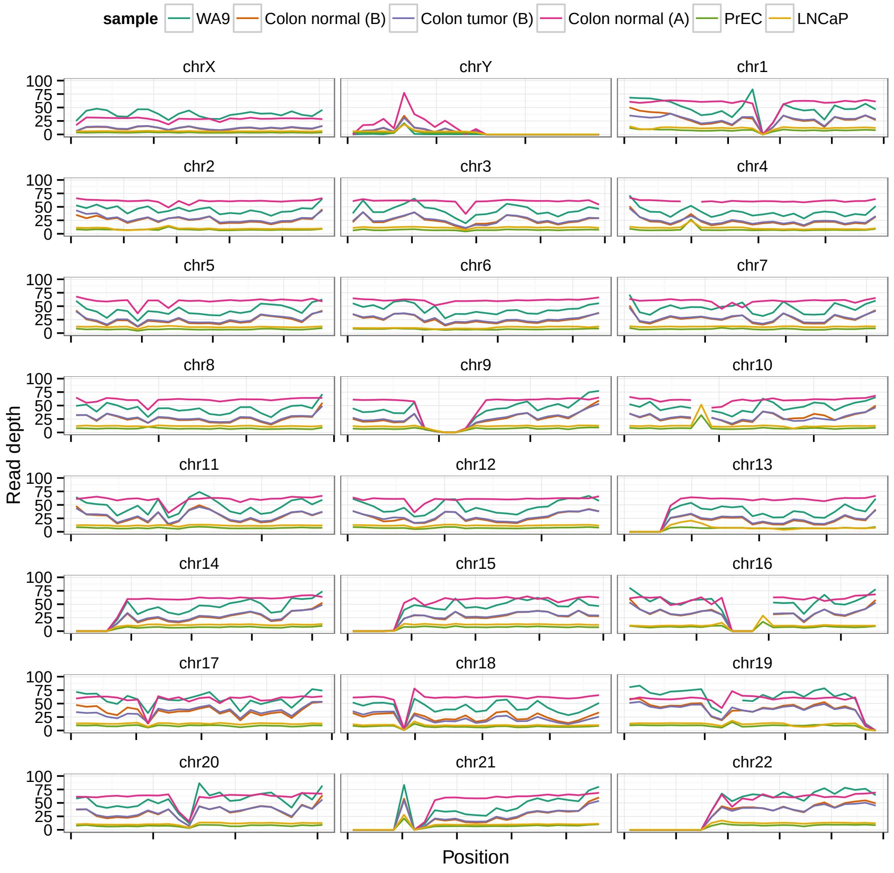

Figure S28 **Read depth for the data used in this paper.** The figure shows the read depth for each position throughout the genome for the six samples used in this paper (see Table 4 in the main text). Read depth is generally very low for LNCaP (10.1 reads on average per position) and for PrEC (6.8 reads). Also read length differs: it is approximately 100 bp for WA9, the three colon samples and LNCaP but only 75 bp for PrEC (see Table 4 in the main text).
